# Supplementary material for: Pharmacogenetics to Avoid Loss of Hearing (PALOH) trial: a protocol for a prospective observational implementation trial
Source: BMJ Open. 2021 Jun 16;11(6):e044457. doi: 10.1136/bmjopen-2020-044457 (PMC8211036; doi:10.1136/bmjopen-2020-044457)
Supplement: Author's manuscript [file bmjopen-2020-044457.draft_revisions.pdf]

BMJ Open

BMJ Open is committed to open peer review. As part of this commitment we make the peer review history of every article we publish publicly available.

When an article is published we post the peer reviewers' comments and the authors' responses online. We also post the versions of the paper that were used during peer review. These are the versions that the peer review comments apply to.

The versions of the paper that follow are the versions that were submitted during the peer review process. They are not the versions of record or the final published versions. They should not be cited or distributed as the published version of this manuscript.

BMJ Open is an open access journal and the full, final, typeset and author-corrected version of record of the manuscript is available on our site with no access controls, subscription charges or pay-per-view fees (http://bmjopen.bmj.com).

If you have any questions on BMJ Open's open peer review process please email info.bmjopen@bmj.com

BMJ Open

The Pharmacogenetics to Avoid Loss of Hearing (PALOH) Trial: A Protocol for a Prospective Observational Implementation Trial

Journal:	*BMJ Open*
Manuscript ID	bmjopen-2020-044457
Article Type:	Protocol
Date Submitted by the Author:	14-Sep-2020
Complete List of Authors:	McDermott, John; Manchester University NHS Foundation Trust, Manchester Centre for Genomic Medicine ; The University of Manchester, Division of Evolution and Genomic Sciences<br>Mahood, Rachel; Manchester University NHS Foundation Trust, Manchester Centre for Genomic Medicine<br>Stoddard, Duncan; DS Analytics and Machine Learning Ltd; Manchester University NHS Foundation Trust, Manchester Centre for Genomic Medicine<br>Mahaveer, Ajit; Manchester University NHS Foundation Trust, Neonatal Intensive Care Unit<br>Turner, Mark; Liverpool Women's Hospital NHS Foundation Trust<br>Corry, Rachel; Manchester University NHS Foundation Trust, Manchester Centre for Genomic Medicine<br>Garlick, Julia; Manchester University NHS Foundation Trust, Manchester Centre for Genomic Medicine<br>Miele, Gino; Genedrive plc<br>Ainsworth, Shaun; Genedrive plc<br>Kemp, Laura; Genedrive plc<br>Bruce, Iain; Manchester University Hospitals NHS Foundation Trust, Paediatric ENT Department<br>Body, Richard; Manchester University NHS Foundation Trust, Emergency Department; The University of Manchester, Division of Cardiovascular Sciences<br>Ulph, Fiona; University of Manchester<br>Macleod, Rhona; Manchester University NHS Foundation Trust, Manchester Centre for Genomic Medicine<br>Harvey, Karen; Liverpool Women's Hospital NHS Foundation Trust<br>Booth, Nicola; Manchester University NHS Foundation Trust, Neonatal Intensive Care Unit<br>Roberts, Peter; Market Access & Reimbursement Solutions<br>Wilson, Paul; University of Manchester, Alliance Manchester Business School<br>Newman, William; Manchester University NHS Foundation Trust, Manchester Centre for Genomic Medicine; University of Manchester, Division of Evolution and Genomic Sciences
Keywords:	Neonatal intensive & critical care < INTENSIVE & CRITICAL CARE, GENETICS, Audiology < OTOLARYNGOLOGY, Adverse events < THERAPEUTICS, PREVENTIVE MEDICINE

I, the Submitting Author has the right to grant and does grant on behalf of all authors of the Work (as defined in the below author licence), an exclusive licence and/or a non-exclusive licence for contributions from authors who are: i) UK Crown employees; ii) where BMJ has agreed a CC-BY licence shall apply, and/or iii) in accordance with the terms applicable for US Federal Government officers or employees acting as part of their official duties; on a worldwide, perpetual, irrevocable, royalty-free basis to BMJ Publishing Group Ltd ("BMJ") its licensees and where the relevant Journal is co-owned by BMJ to the co-owners of the Journal, to publish the Work in this journal and any other BMJ products and to exploit all rights, as set out in our licence.

The Submitting Author accepts and understands that any supply made under these terms is made by BMJ to the Submitting Author unless you are acting as an employee on behalf of your employer or a postgraduate student of an affiliated institution which is paying any applicable article publishing charge ("APC") for Open Access articles. Where the Submitting Author wishes to make the Work available on an Open Access basis (and intends to pay the relevant APC), the terms of reuse of such Open Access shall be governed by a Creative Commons licence – details of these licences and which Creative Commons licence will apply to this Work are set out in our licence referred to above.

Other than as permitted in any relevant BMJ Author's Self Archiving Policies, I confirm this Work has not been accepted for publication elsewhere, is not being considered for publication elsewhere and does not duplicate material already published. I confirm all authors consent to publication of this Work and authorise the granting of this licence.

For peer review only

The Pharmacogenetics to Avoid Loss of Hearing (PALOH) Trial: A Protocol for a Prospective Observational Implementation Trial

McDermott JH[1,2†], Mahood R [1,2], Stoddard D[3], Mahaveer A[4], Turner M[5], Corry R[1], Garlick J[1], Miele G[6], Ainsworth S[6], Kemp L[6], Bruce IA[7,8] Body R[9,10], Ulph F[11], MacLeod R[1], Harvey K[5], Booth N[4], Roberts P[12] Wilson P[13], Newman WG[1,2†]

1. Manchester Centre for Genomic Medicine, St Mary's Hospital, Manchester University NHS Foundation Trust, Manchester, M13 9WL, UK
2. Division of Evolution and Genomic Sciences, School of Biological Sciences, University of Manchester, Manchester, UK
3. DS Analytics and Machine Learning Ltd, 12 Hammersmith Grove, Hammersmith, London, M6 7AP
4. Newborn Intensive Care Unit, Manchester University NHS Foundation Trust, Manchester, M13 9WL, UK
5. Newborn Intensive Care Unit, Liverpool Women's Hospital, Liverpool Crown St, Liverpool L8 7SS, UK
6. Genedrive Diagnostics Ltd, 48 Grafton Street, Manchester, M13 9XX
7. Hearing Health Theme Manchester NIHR Biomedical Research Centre, Manchester, UK
8. Pediatric ENT Department, Royal Manchester Children's Hospital, Manchester University NHS Foundation Trust, Manchester, United Kingdom, M13 9WL, UK
9. Emergency Department, Manchester University NHS Foundation Trust, Manchester, M13 9WL, UK
10. Division of Cardiovascular Sciences, University of Manchester, Manchester, UK
11. Division of Psychology & Mental Health, School of Health Sciences, Faculty of Biology, Medicine and Health, University of Manchester, Manchester Academic Health Science Centre, Oxford Road, Manchester M13 9PL, UK
12. Market Access & Reimbursement Solutions Ltd, 43 Castle Street, Liverpool, Merseyside
13. Alliance Manchester Business School, University of Manchester, Manchester, UK

†Correspondence to Professor William Newman & Dr John Henry McDermott, Manchester Centre for Genomic Medicine, Manchester University Hospitals NHS Foundation Trust, Manchester M13 9WL, UK

Tel: 0161 276 6506
e-mail: william.newman@manchester.ac.uk & john.mcdermott2@mft.nhs.uk

Funding support through an NIHR i4i program award (II-LB-0417-20002), with additional support from the Manchester NIHR Biomedical Research Centre, Hearing Health Theme (IS-BRC-1215-20007). Pilot data support from Action on Hearing Loss Flexi grant F67_Newm.

Running Title: Point of care genetic testing to prevent hearing loss

Word Count (Including References): 3482

Keywords: Pharmacogenetics, point-of-care, gentamicin, hearing-loss, neonates, genetics

For peer review only - http://bmjopen.bmj.com/site/about/guidelines.xhtml

Abstract

Introduction: In conjunction with a beta-lactam, aminoglycosides are the first-choice antibiotic for empirical treatment of sepsis in the neonatal period. The m.1555A>G variant predisposes to ototoxicity after aminoglycoside administration and has a prevalence of 1 in 500. Current genetic testing can take over 24-hours, an unacceptable delay in the acute setting. This prospective-observational trial will implement a rapid point of care test (POCT), facilitating tailored antibiotic prescribing to avoid hearing loss.

Methods: The genedrive™ POCT can detect the m.1555A>G variant in 26 minutes from buccal swab. This system will be integrated into the clinical pathways at two large UK neonatal centres over a minimum 6-month period. The primary outcome is the number of neonates successfully tested for the variant out of all babies prescribed antibiotics. As a secondary outcome, clinical timings will be compared to data collected prior to implementation, measuring the impact on routine practice.

Ethics and Dissemination: An opt-out consenting process was approved by the Research Ethics Committee (REC) in August 2019. Results will be published in full on completion of the study.

Discussion: The m.1555A>G variant is associated with aminoglycoside induced ototoxicity. This was established in 1993, yet technology has not been available to rapidly test for the variant. The genedrive™ assay can detect the variant in a practice-changing timeframe. However, it has not been established whether such a test could be practically implemented. To our knowledge, this trial represents the first example of a genetic POCT being employed in the neonatal setting to alter management acutely.

For peer review only - http://bmjopen.bmj.com/site/about/guidelines.xhtml

**Title**	Pharmacogenetics to Avoid Loss of Hearing (PALOH): Study Protocol for a Prospective Observational Implementation Trial
**Trial registration**	ISRCTN13704894
**Protocol version**	V 1.3
**Funding**	NIHR Invention for Innovation (i4i) Programme. Grant Number II-LB-0417-20002
**Name and contact information for the trial sponsor**	Manchester University NHS Foundation Trust<br>Dr Lynne Webster<br>Research Office<br>Manchester University NHS Foundation Trust<br>Oxford Road<br>Manchester<br>M13 9PL<br>Lynne.webster@mft.nhs.uk<br>Tel: 01612764125<br>Fax: 01612765766
**Role of sponsor**	The Sponsor, Manchester University NHS Foundation Trust, takes overall responsibility for proportionate, effective arrangements being in place to set up, run and report this research project. Sponsorship responsibilities include; ensuring suitability of the research proposal and researchers involved, facilitating peer review, promoting research transparency, ensuring that the necessary regulatory approvals are in place prior to study start, that there is adequate funding for the duration of the research and that effective arrangements are in place for reporting and monitoring the research. The Trust aims to support MFT staff to initiate and undertake research with our patients and staff.

For peer review only

For peer review only - http://bmjopen.bmj.com/site/about/guidelines.xhtml

Strengths and Limitations

- This manuscript describes a prospective observational implementation trial of a rapid POCT to detect the m.1555A>G before antibiotic administration in the acute NICU setting.
- To our knowledge this is the first example of a genetic POCT being implemented in the acute neonatal setting to alter management, therefore we have addressed several novel ethical and practical challenges.
- Given the implementation of the work, the primary outcome is not related to the avoidance of hearing loss or detection of the variant, rather how well the NICUs are able to use the technology.
- Given the relatively rare nature of the variant (1 in 500), it may transpire that the variant is never detected throughout the study, meaning some secondary outcomes cannot be reliably assessed.

For peer review only

1. Background

Aminoglycosides are broad-spectrum antibiotics which act by binding to the 16S rRNA component of the bacterial 30S ribosomal subunit, resulting in the translation of truncated proteins.[1; 2] These abnormal proteins stimulate a stress response within the bacteria culminating in cell death. Due to their low cost and effectiveness, they are one of the most frequently prescribed medicines globally.[3] The National Institute for Health and Care Excellence (NICE) advises the use of intravenous benzylpenicillin with gentamicin as the first-choice antibiotic regimen for empirical treatment of infection in the neonatal period (NICE CG149). This combination has the major advantage of having a narrow spectrum of activity and lower risk of antibiotic resistance compared to alternative antibiotic regimens, such as third generation cephalosporins which are recommended as second line agents.

The side effect profile from protracted courses of aminoglycosides is well known, with nephrotoxicity, vestibulotoxicity and ototoxicity commonly recognised in cohorts who receive large amounts of aminoglycoside.[4] Careful analysis revealed that aminoglycoside induced ototoxicity (AIO) clusters within families and this trait seemed to be transmitted maternally, suggesting mitochondrial inheritance. Further work demonstrated that this susceptibility was caused by a variant in the 12S rRNA (RNR1) m.1555A>G.[5] This causes a change in the conformation of the 12S rRNA, producing a structure more like the bacterial 16S rRNA.

The m.1555A>G variant is strongly associated with AIO and has a reported prevalence of 0.2% (~1 in 500).[6] It has previously been suggested that genetic testing should be used in children requiring aminoglycosides to prevent hearing loss and that this approach would be cost-effective when balanced against the costs of lifelong deafness.[3; 6] In many centers, children with cystic fibrosis are tested for the variant at diagnosis as it is expected that these individuals will require aminoglycoside antibiotics at some stage in their lives. This is typically undertaken by a validated genotyping assay in an accredited diagnostic laboratory, a strategy which takes approximately 3-4 days to return a result.

For peer review only

Understandably, this testing strategy is not suitable for use in clinical settings where administration of antibiotics is required within an hour of the decision to start treatment with antibiotics.

Currently there is an inability to test for the m.1555A>G variant in the acute setting such as babies admitted to the neonatal intensive care unit (NICU), representing approximately 90,000 admissions per year in the UK alone. By introducing a simple genetic point of care test (POCT) with a result in under 30 minutes, we have the potential to avoid approximately 180 cases of severe/profound irreversible deafness every year. Those babies with the m.1555A>G variant could be prescribed an equally effective second line antibiotic, such as a cephalosporin.

Alongside an industry partner, genedrive™, we have a developed a small and robust thermocycler platform for rapid point-of-care diagnostic testing of the m.1555A>G variant which is comparable to gold-standard genotyping assay and has been CE certified for clinical use. This protocol describes a multi-centre prospective observational trial to assess the implementation of the POCT in the clinical setting.

2. Methods

2.1 Study Design

Investigator-initiated, multi-centre, prospective-observational, implementation trial. Two study sites, both of which are recruiting and experimental.

2.2 Primary Objective

To critically assess the performance of a newly developed genetic POCT for use on the NICU and measure whether the clinical teams can integrate the test into their clinical practice without disrupting normal standard of care, facilitating tailored antibiotic prescribing.

2.3 Secondary Objectives

Exploratory objectives include an assessment of the wider impact of the implementation, including impact on resources and clinical timelines. We will also assess instances where the assay was not used and consider reasons for deviation. The reliability of the assay itself will also be measured through

For peer review only - http://bmjopen.bmj.com/site/about/guidelines.xhtml

regular monitoring of results, assessment of test failures and confirmation against gold-standard genotyping.

2.4 Study Centres and Time Schedule

Participants will be recruited from two large UK based NICUs in the UK, Manchester University NHS Foundation Trust (MFT) and Liverpool Women's NHS Foundation Trust (LWH). Both sites will recruit participants and conduct the trial. The trial will start in January 2020 and it is expected that the last participant will be recruited in November 2020.

2.5 Primary Outcome

The primary outcome is the number of neonates who are successfully tested for the m.1555A>G variant via this novel POCT genetic test as a proportion of all babies who receive aminoglycoside antibiotics on the two participating NICUs.

2.6 Secondary Outcomes

This trial will also assess several secondary outcomes including the total number of neonates identified with the m.1555A>G genetic variant (positive result) as a proportion of the study population. An assessment of resource impact will be undertaken, including a health economic analysis of additional staff time to secure sample and testing and costs associated with alternative antibiotic prescribing for any positive tests. We will record the number of neonates where testing was not undertaken and the rationale for this. Correlation of the POCT result with the current in-house reference assay will be assessed and any test fails will be reported. Finally, we will assess the variance in timings of antibiotic therapy between "standard of care" (before implementation) and following implementation.

2.7 Inclusion criteria

All babies admitted to NICU across the two participating sites, commencing from the trial start date. This includes babies admitted directly from delivery suite, midwifery led units or transferred from another neonatal unit. At the LWH site, babies who are screened for infection on the NICU but then

For peer review only - http://bmjopen.bmj.com/site/about/guidelines.xhtml

transferred to external wards (not formally admitted to NICU) will also be included. It is a clinical decision whether the babies are being screened for infection as part of their assessment, reflecting the real-world, pragmatic, nature of the trial. This also reflects the variation in admission procedures between the two trial sites where pathways differ due to local practices.

Given that all babies admitted to NICU are eligible for recruitment, some of those recruited will not go onto receive antibiotic therapy. If the baby did not go onto receive antibiotics, their data will still form part of the dataset for analysis.

2.8 Exclusion Criteria

Babies requiring antibiotics immediately, as determined by the admitting clinician, with already established intravenous (IV) access. These exclusion criteria recognize the potential urgency of any decision to deliver antibiotics. If IV access has not been achieved, then this may provide time to run the assay.

2.9 Training

Training of a minimum of 80% of relevant nursing and medical staff within the two NICUs will commence in the 6 months prior to study start. This figure has been chosen, based on guidance from senior NICU clinicians and nursing staff, to ensure that there will always be staff members on-shift who are trained to undertake the assay throughout study.

Training will include practical use and interpretation of the assay, with Standard Operating Procedures for use integrated into the standard admission procedure. A “train the trainer” approach will be adopted, where a number of experienced NICU research nurses plus additional clinical nursing staff identified as “super-users” will receive training directly from representatives of the device manufacturer. These designated super-users will then cascade training to the remaining nursing and medical staff within the two neonatal services.

For peer review only

Ongoing refresher training and online resources will be made available throughout the trial. A training log will be maintained detailing staff competency including dates of training, in order to identify any correlation of assay performance with increasing training numbers. NICU research nurses and co-investigators will be fully Good Clinical Practice (GCP) competent. A local, named co-investigator (neonatal consultant) will be responsible for clinical staff performing any duties in accordance with the protocol, GCP and local requirements.

2.10 Testing Process & Assessment

Participants will be recruited as they are admitted or reviewed by the NICU team. Given the acute nature of the project and the timeframes involved, there is no opportunity to undertake a formal pre-trial screening assessment or prospective consenting process. All babies admitted to NICU or screened for infection by the NICU team will be deemed eligible for recruitment unless there is a clinical decision by the responsible admitting clinician that the exclusion criterion is met and the individual requires immediate antibiotic therapy, without delay.

Following recruitment, the time that a decision was made to prescribe antibiotics represents time 0 (Figure 1). If antibiotics were not prescribed, then time 0 is represented by the time of admission. The genetic POCT is performed from a buccal swab. When babies are admitted to NICU several swabs are already taken at admission. Where babies are recruited to the study, the m.1555A>G buccal swab will be performed alongside these normal admission processes. The assay will be commenced, and normal practice will continue. Once the assay result is available, the result can be used to then undertake personalized antibiotic prescribing.

As part of the study the following variables will be recorded; participant demographics, admission time, time of decision to prescribe, time swab taken, time assay started, time of assay result, assay result, time of antibiotic prescription, antibiotics prescribed. Where a positive result is identified and antibiotic therapy is required, an alternative antibiotic regimen (standard non-gentamicin pathway as per hospital guidelines) will be prescribed and a referral should be made to clinical genetics for familial

For peer review only - http://bmjopen.bmj.com/site/about/guidelines.xhtml

cascade testing. Where there is a test-fail, standard of care should be followed, i.e the baby should be administered gentamicin if clinically indicated.

Assay performance will be assessed throughout the study, with 10% of samples each month tested via gold-standard pyrosequencing. Any positive samples will also be confirmed as per standard clinical pathways. Any divergence between the POCT and gold-standard methodology will be reviewed by the trial management committee.

Data collection will be undertaken contemporaneously by the clinical staff and any missing data will be collected by the NICU research nurses. Data will be entered into an electronic database by the clinical team and a study ID will be generated. The participants will subsequently be anonymized to the research team. If an individual is not tested for any reason (e.g. test overlooked, staff availability or the individual meets the exclusion criteria), these incidences will be captured retrospectively by NICU research nurses by checking daily admission records and the reasons will be documented.

2.11 Samples Size

The sample size (approximately 900 babies) is estimated based upon the expected number of admissions across both clinical sites over an approximate 10-month trial period. To achieve a statistical power of 90% with a 5% significance level, a margin of 0.5 (in Cohen's d, which equates to about 7 minutes difference given the standard deviation in the control sample) and a control sample of N = 50, a test sample size of 112 is required. With the forecast test sample size of 900, statistical power should be around 95% to detect variance in clinical timings before and after implementation.

2.12 Statistical Analysis

For the primary outcome, simple descriptive statistics will be used to provide a metric for the proportion of babies tested for the variant over the number who were ultimately prescribed aminoglycoside antibiotics.

For peer review only

Regarding the secondary outcome measures, the objective is to establish that the introduction of the POCT does not significantly impact the time between admission to NICU and the prescription of antibiotics. The burden of proof rests on the research hypothesis, meaning the null hypothesis is that the new POCT does impact this quantity. This is a so-called non-inferiority significance test. The quantity of interest is the mean difference between the admission to ICU and prescription of antibiotics timestamps in two situations: with and without the POCT test $(\mu_c, \mu_{t)})$. The null hypothesis is that the difference between these quantities is greater than or equal to some prespecified margin (Δ) i.e. $H_0: \mu_c - \mu_t \geq \Delta; H_A: U_t - U_c < \Delta$. A type I error here is concluding the time durations are equivalent when in fact with the POCT is worse.

The two samples are of unequal size, with the control ('before implementation') sample having about n_c=50, and a forecasted test sample size of around n_t=900. We also have no insight into population variance and cannot assume they are the same in both groups (it may for example take some staff members longer to carry out the new test than others). As such, a one-sided Welch t-test will be used to reject or not reject the null hypothesis.

Throughout the study we anticipate that there will be iterative updates to the POCT technology, as avenues for optimization emerge because of our findings from real-world implementation. The timings of these updates will be recorded and separate post-hoc statistical analysis will be undertaken to assess the impact of each update.

2.13 Study Organisation

Two groups have been convened to oversee the trial, a management group, and a steering committee. The Trial Management Group convenes monthly to ensure all practical details of the trial are progressing well, within the agreed milestones, and is led by the study Chief Investigator. The trial management group will act as the data monitoring committee. Membership is independent of the sponsors. The data management team will have access to anonymized data and will review assay

For peer review only - http://bmjopen.bmj.com/site/about/guidelines.xhtml

performance and trial conduct on a monthly basis- specifically the concordance with gold standard. All data will be analyzed by an independent statistical team who report monthly to the trial management group. Any amendments to the protocol will be communicated to the REC, funding agency and the trials registry.

The Trial Steering Committee meets on a 6 monthly basis to monitor the performance of the trial against the agreed project plan and advise on scientific and technical aspects of the project. The membership is independent of the Sponsor and Investigators.

A Stakeholders group has also been assembled to advise on the design and dissemination of the study intervention in the interests of the intended end users and participants. This membership includes personnel from key clinical services (neonatology and paediatric audiology), commissioners (NHS England) and newborn and hearing loss charities.

2.14 Patient and Public Involvement

Involvement of parent and public representatives has been a critical component of the development of this trial protocol. Parent representatives are involved in both the trial management groups, stakeholders committee and a separate Public and Patient Involvement and Engagement (PPIE) panel, providing both neonatal care and/or hearing loss experience. The PPIE panel was involved in the development of the both protocol and ethics application. The PI and co-investigators presented early versions of the texts to the PPIE panel and any potentially contentious issues were discussed in more detail. The insights gained from these meetings were used to refine the applications prior to submission.

2.15 Ethics and Adverse Events

This is the world's first trial, that we are aware of, of a genetic POCT to personalize medicine in the acute neonatal setting. As such, there have been several ethical challenges, mainly performing DNA analysis without qualifying consent. Prospective consent is not possible given the acute nature of the

For peer review only - http://bmjopen.bmj.com/site/about/guidelines.xhtml

admission, therefore we sought permission to proceed with an "opt-out" consenting process. The opt-out consenting process in relation to this study have been described in detail previously.[7] Approval was provided by the Research Ethics Committee (REC) and the Human Research Authority (HRA) in August 2019. We will record any adverse event related to the sample collection procedure or directly related to the testing process.

2.16 Dissemination of Results

The results of the study will be submitted to international peer-reviewed scientific journals, irrespective of their outcome and presented as per best practice STROBE guidelines for observational trials.

2.17 Funding

Funding support through an NIHR i4i program award (Grant Number: II-LB-0417-20002), with additional support from the Manchester NIHR Biomedical Research Centre, Hearing Health Theme (IS-BRC-1215-20007). Pilot data support from Action on Hearing Loss Flexi grant F67_Newm.

3. Discussion

To our knowledge, the PALOH study represents the first trial of a genetic POCT designed to alter management in an acute, time-sensitive setting. Successful implementation could avoid up to 180 cases of aminoglycoside induced ototoxicity in the UK each year, and many more worldwide. However, this study has implications beyond this gene-drug pair. The anticipated results will indicate whether genetic technology can be implemented in the acute setting more broadly, a hitherto untested concept. The unprecedented nature of this work led to several practical and ethical challenges in the design of the protocol.

Several studies have previously reported that most clinicians are not confident with the interpretation and implementation of genetic data.[8-10] With this in mind, we ensured that the assay implemented as part of this trial was as straightforward to interpret as possible, providing a binary readout for the presence or absence of the m.1555A>G variant. However, even if an assay is simple to operate and

For peer review only - http://bmjopen.bmj.com/site/about/guidelines.xhtml

For peer review only

interpret, we recognized that the imposition of any alien process in such a critical time-period could impact adoption. We therefore designed this protocol to include a comprehensive training strategy prior to the trial commencing on both units. This significant undertaking ensures that over 500 health care professionals, greater than 80% of all staff, have exposure to the system before implementation.

Due to CE certification requirements for medical devices in the European Union (EU), any new system must be validated prior to clinical implementation. For a technology which has been designed for use in the acute setting, this requirement poses unique challenges. Although we were able to thoroughly assess the technology in the pre-clinical laboratory setting, satisfying the requirements for CE certification, it was not possible to perform analytical validation in its intended use setting, the NICU. This was because any result generated in this setting would be actionable and could impact management, precipitating an ethical dilemma. As a result, we determined that the most appropriate strategy was to regularly review the performance of the assay following implementation and introduce any updates to the training or technology as required. We have therefore made clear in our analysis plan that outcome statistics will be reported for the study as a whole and for each time-period post-update, if any such updates are necessary.

Given the acute context in which the PALOH trial is taking place, it was recognised that it would not be practicable for informed consent to be gained prospectively. Newborns admitted to NICU are, by definition, acutely unwell and it is likely to represent one of the most stressful time in a parent’s life.

As such, it was determined that asking for consent to perform this genetic test immediately after birth would be inappropriate. Following discussion with stakeholders including parents, neonatologists, and geneticists, it was decided that an opt-out consenting model should be employed. Our PPIE panel were particularly helpful during this process, providing a forum to discuss the various consenting options where we could consider relative benefits and costs of each strategy.

For peer review only

Neonates are tested for the variant as part of their admission process via a buccal swab, the variant details then used to inform prescribing and data is subsequently gathered and analysed. Parents will be provided information regarding the trial by the NICU team as part of an existing parental information pack. They would have the right to ask for their child's data to be removed from the study at that stage. This provides a mechanism to opt-out prior to the analysis stage although, critically, by this point their child will have already had tailored antibiotic prescribing based on the presence or absence of the variant.

To our knowledge this represents the first example of an opt-out consenting model being used in a trial involving genetic testing. In the summer of 2019, an application for ethical approval was submitted to the Health Research Authority Research Ethics Service (RES), proposing that the importance of this genetic test and the potential benefits warranted approval of an opt-out consenting model. After consideration, the REC approved the PALOH trial design in full pending HRA approval, making it the first trial of a genetic POCT to alter management in the acute setting. However, in the week following the REC meeting the PALOH study team received notice that the HRA were withholding approval as they had concerns that the study was in violation of the Human Tissue Act (2004) (HT Act). Specifically, the HRA felt that the trial design did not meet the legal definition of "qualifying consent" as outlined in the HT Act.

Following protracted ethical and legal discussion, the trial design was approved following the agreement that the act of performing the initial test represents a clinical decision, rather than a research question. This approach was then permissible under Schedule 4 Part 2 of the HT Act which outlines that "the medical diagnosis or treatment of the person whose body manufactured the DNA" is an excepted purpose for DNA analysis without formal consent.[7] We believe that the PALOH study represents the first time this ethicolegal issue has been considered. As such, our methodology may be of value to future clinical academics exploring the application of similar pharmacogenetic biomarkers.

For peer review only - http://bmjopen.bmj.com/site/about/guidelines.xhtml

The anticipated results of the PALOH trial will assess whether a genetic POCT can be used to tailor antibiotic prescribing in the acute NICU setting. This will be of value not only in avoiding AIO but also in optimising management in other disease areas, as the relevance of genetics in everyday clinical practice continues to develop. We believe that this trial methodology and the ethical issues outlined in this work will be of value when researchers are designing similar studies in the future.

4. Trial Status

The PALOH Study is currently ongoing. Recruitment began in January 2020 and is anticipated to be completed in November 2020.

5. Declarations

Ethics Approval: Health Research Authority (HRA) and Health and Care Research Wales (HCRW) approval was granted by the Research Ethics Committee at Liverpool Women's Hospital on the 23rd of September 2019. REC Reference 19/NW/0400.

Consent for Publication: Consent for publication is not applicable as there are no identifying images or other personal details of participants presented in this protocol manuscript.

Competing Interests: SA, LK and GM are employees of genedrive. The other authors report no competing interests.

Data: All results of this trial will be submitted for peer reviewed publication. Final data will be submitted to an open access repository on publication of the final trial manuscript.

Author Statements: JHM and WGN developed the concept for the study. SA, LK and GM designed and optimized the assay for implementation. All authors contributed to the development of the protocol led by AM and MT and the MFT and LWH sites, respectively. KH and NB manage the data collection at each clinical site. RM is research manager for PALOH. DS, PR, and PW are involved in data analysis. RMc and FU have designed and undertaken stakeholder interviews which contribute to the design of the protocol. JG and RC, along with RM, have overseen the PPIE process. RB and PW have advised on the implementation process and broader study design. All authors are responsible for either the recruitment of participants, data collection or data analysis. All authors read and approved the final version of this manuscript.

For peer review only - http://bmjopen.bmj.com/site/about/guidelines.xhtml
For peer review only

Acknowledgements: The authors would like to thank the patients and family members who took part in the study. We would like to acknowledge the huge efforts of the nurses, physicians and administrative staff at both study sites who have been instrumental in developing the project. We are very grateful to the parents and public representatives who sat on the PPIE committee and advised on the development of the project.

Figure Legends

Figure 1. Flow diagram demonstrating the admission, testing, and prescribing process in the PALOH Trial. The time that a decision was made to prescribe antibiotics represents time 0. If antibiotics were not prescribed, then time 0 is represented by the time of admission.

For peer review only

For peer review only - http://bmjopen.bmj.com/site/about/guidelines.xhtml

References

1. Schacht, J., Talaska, A.E., and Rybak, L.P. (2012). Cisplatin and aminoglycoside antibiotics: hearing loss and its prevention. Anat Rec (Hoboken) 295, 1837-1850.
2. Lanvers-Kaminsky, C., and Ciarimboli, G. (2017). Pharmacogenetics of drug-induced ototoxicity caused by aminoglycosides and cisplatin. Pharmacogenomics 18, 1683-1695.
3. Bitner-Glindzicz, M., and Rahman, S. (2007). Ototoxicity caused by aminoglycosides. BMJ 335, 784-785.
4. Prayle, A., and Smyth, A.R. (2010). Aminoglycoside use in cystic fibrosis: therapeutic strategies and toxicity. Curr Opin Pulm Med 16, 604-610.
5. Prezant, T.R., Agapian, J.V., Bohlman, M.C., Bu, X., Oztas, S., Qiu, W.Q., Arnos, K.S., Cortopassi, G.A., Jaber, L., and Rotter, J.I. (1993). Mitochondrial ribosomal RNA mutation associated with both antibiotic-induced and non-syndromic deafness. Nat Genet 4, 289-294.
6. Bitner-Glindzicz, M., Pembrey, M., Duncan, A., Heron, J., Ring, S.M., Hall, A., and Rahman, S. (2009). Prevalence of mitochondrial 1555A-->G mutation in European children. N Engl J Med 360, 640-642.
7. McDermott, J.H. (2020). Genetic testing in the acute setting: a round table discussion. J Med Ethics.
8. Christensen, K.D., Vassy, J.L., Jamal, L., Lehmann, L.S., Slashinski, M.J., Perry, D.L., Robinson, J.O., Blumenthal-Barby, J., Feuerman, L.Z., Murray, M.F., et al. (2016). Are physicians prepared for whole genome sequencing? a qualitative analysis. Clin Genet 89, 228-234.
9. Hauser, D., Obeng, A.O., Fei, K., Ramos, M.A., and Horowitz, C.R. (2018). Views Of Primary Care Providers On Testing Patients For Genetic Risks For Common Chronic Diseases. Health Aff (Millwood) 37, 793-800.
10. Selkirk, C.G., Weissman, S.M., Anderson, A., and Hulick, P.J. (2013). Physicians' preparedness for integration of genomic and pharmacogenetic testing into practice within a major healthcare system. Genet Test Mol Biomarkers 17, 219-225.

For peer review only - http://bmjopen.bmj.com/site/about/guidelines.xhtml

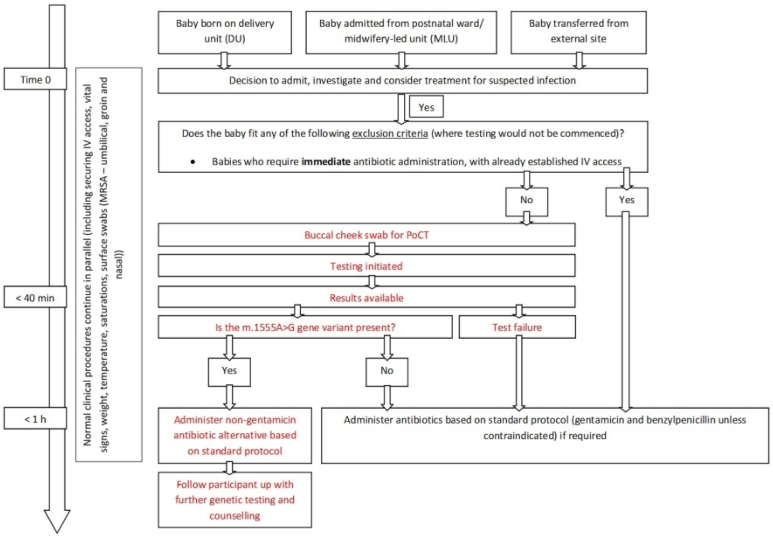

Figure 1. Flow diagram demonstrating the admission, testing, and prescribing process in the PALOH Trial. The time that a decision was made to prescribe antibiotics represents time 0. If antibiotics were not prescribed, then time 0 is represented by the time of admission.

287x174mm (150 x 150 DPI)

STROBE Statement—checklist of items that should be included in reports of observational studies

	Item No	Recommendation	Page No
**Title and abstract**	1	(*a*) Indicate the study's design with a commonly used term in the title or the abstract	1
		(*b*) Provide in the abstract an informative and balanced summary of what was done and what was found	2
**Introduction**			
Background/rationale	2	Explain the scientific background and rationale for the investigation being reported	5
Objectives	3	State specific objectives, including any prespecified hypotheses	6
**Methods**			
Study design	4	Present key elements of study design early in the paper	6
Setting	5	Describe the setting, locations, and relevant dates, including periods of recruitment, exposure, follow-up, and data collection	7
Participants	6	(*a*) *Cohort study*—Give the eligibility criteria, and the sources and methods of selection of participants. Describe methods of follow-up *Case-control study*—Give the eligibility criteria, and the sources and methods of case ascertainment and control selection. Give the rationale for the choice of cases and controls *Cross-sectional study*—Give the eligibility criteria, and the sources and methods of selection of participants	7 & 8
		(*b*) *Cohort study*—For matched studies, give matching criteria and number of exposed and unexposed *Case-control study*—For matched studies, give matching criteria and the number of controls per case	N/A
Variables	7	Clearly define all outcomes, exposures, predictors, potential confounders, and effect modifiers. Give diagnostic criteria, if applicable	7
Data sources/ measurement	8*	For each variable of interest, give sources of data and details of methods of assessment (measurement). Describe comparability of assessment methods if there is more than one group	*9*
Bias	9	Describe any efforts to address potential sources of bias	13
Study size	10	Explain how the study size was arrived at	9 & 10
Quantitative variables	11	Explain how quantitative variables were handled in the analyses. If applicable, describe which groupings were chosen and why	10
Statistical methods	12	(*a*) Describe all statistical methods, including those used to control for confounding	10
		(*b*) Describe any methods used to examine subgroups and interactions	N/A
		(*c*) Explain how missing data were addressed	N/A
		(*d*) *Cohort study*—If applicable, explain how loss to follow-up was addressed *Case-control study*—If applicable, explain how matching of cases and controls was addressed *Cross-sectional study*—If applicable, describe analytical methods taking account of sampling strategy	N/A
		(*e*) Describe any sensitivity analyses	N/A

For peer review only - http://bmjopen.bmj.com/site/about/guidelines.xhtml

**Results**			
Participants	13*	(a) Report numbers of individuals at each stage of study—eg numbers potentially eligible, examined for eligibility, confirmed eligible, included in the study, completing follow-up, and analysed	Awaited
		(b) Give reasons for non-participation at each stage	Awaited
		(c) Consider use of a flow diagram	Awaited
Descriptive data	14*	(a) Give characteristics of study participants (eg demographic, clinical, social) and information on exposures and potential confounders	Awaited
		(b) Indicate number of participants with missing data for each variable of interest	Awaited
		(c) *Cohort study*—Summarise follow-up time (eg, average and total amount)	Awaited
Outcome data	15*	*Cohort study*—Report numbers of outcome events or summary measures over time	Awaited
		Case-control study—Report numbers in each exposure category, or summary measures of exposure	Awaited
		Cross-sectional study—Report numbers of outcome events or summary measures	Awaited
Main results	16	(*a*) Give unadjusted estimates and, if applicable, confounder-adjusted estimates and their precision (eg, 95% confidence interval). Make clear which confounders were adjusted for and why they were included	Awaited
		(*b*) Report category boundaries when continuous variables were categorized	Awaited
		(*c*) If relevant, consider translating estimates of relative risk into absolute risk for a meaningful time period	Awaited
Other analyses	17	Report other analyses done—eg analyses of subgroups and interactions, and sensitivity analyses	Awaited
**Discussion**			
Key results	18	Summarise key results with reference to study objectives	Awaited
Limitations	19	Discuss limitations of the study, taking into account sources of potential bias or imprecision. Discuss both direction and magnitude of any potential bias	Awaited
Interpretation	20	Give a cautious overall interpretation of results considering objectives, limitations, multiplicity of analyses, results from similar studies, and other relevant evidence	Awaited
Generalisability	21	Discuss the generalisability (external validity) of the study results	Awaited
**Other information**			
Funding	22	Give the source of funding and the role of the funders for the present study and, if applicable, for the original study on which the present article is based	3

*Give information separately for cases and controls in case-control studies and, if applicable, for exposed and unexposed groups in cohort and cross-sectional studies.

Note: An Explanation and Elaboration article discusses each checklist item and gives methodological background and published examples of transparent reporting. The STROBE checklist is best used in conjunction with this article (freely available on the Web sites of PLoS Medicine at http://www.plosmedicine.org/, Annals of Internal Medicine at http://www.annals.org/, and Epidemiology at http://www.epidem.com/). Information on the STROBE Initiative is available at www.strobe-statement.org.

For peer review only

The Pharmacogenetics to Avoid Loss of Hearing (PALOH) Trial: A Protocol for a Prospective Observational Implementation Trial

Journal:	*BMJ Open*
Manuscript ID	bmjopen-2020-044457.R1
Article Type:	Protocol
Date Submitted by the Author:	02-Mar-2021
Complete List of Authors:	McDermott, John; Manchester University NHS Foundation Trust, Manchester Centre for Genomic Medicine ; The University of Manchester, Division of Evolution and Genomic Sciences<br>Mahood, Rachel; Manchester University NHS Foundation Trust, Manchester Centre for Genomic Medicine<br>Stoddard, Duncan; DS Analytics and Machine Learning Ltd; Manchester University NHS Foundation Trust, Manchester Centre for Genomic Medicine<br>Mahaveer, Ajit; Manchester University NHS Foundation Trust, Neonatal Intensive Care Unit<br>Turner, Mark; Liverpool Women's Hospital NHS Foundation Trust<br>Corry, Rachel; Manchester University NHS Foundation Trust, Manchester Centre for Genomic Medicine<br>Garlick, Julia; Manchester University NHS Foundation Trust, Manchester Centre for Genomic Medicine<br>Miele, Gino; Genedrive plc<br>Ainsworth, Shaun; Genedrive plc<br>Kemp, Laura; Genedrive plc<br>Bruce, Iain; Manchester University Hospitals NHS Foundation Trust, Paediatric ENT Department<br>Body, Richard; Manchester University NHS Foundation Trust, Emergency Department; The University of Manchester, Division of Cardiovascular Sciences<br>Ulph, Fiona; University of Manchester<br>Macleod, Rhona; Manchester University NHS Foundation Trust, Manchester Centre for Genomic Medicine<br>Harvey, Karen; Liverpool Women's Hospital NHS Foundation Trust<br>Booth, Nicola; Manchester University NHS Foundation Trust, Neonatal Intensive Care Unit<br>Roberts, Peter; Market Access & Reimbursement Solutions<br>Wilson, Paul; University of Manchester, Alliance Manchester Business School<br>Newman, William; Manchester University NHS Foundation Trust, Manchester Centre for Genomic Medicine; University of Manchester, Division of Evolution and Genomic Sciences
**Primary Subject Heading**:	Genetics and genomics

Secondary Subject Heading:	Genetics and genomics, Paediatrics, Pharmacology and therapeutics
Keywords:	Neonatal intensive & critical care < INTENSIVE & CRITICAL CARE, GENETICS, Audiology < OTOLARYNGOLOGY, Adverse events < THERAPEUTICS, PREVENTIVE MEDICINE

SCHOLARONE™ Manuscripts

I, the Submitting Author has the right to grant and does grant on behalf of all authors of the Work (as defined in the below author licence), an exclusive licence and/or a non-exclusive licence for contributions from authors who are: i) UK Crown employees; ii) where BMJ has agreed a CC-BY licence shall apply, and/or iii) in accordance with the terms applicable for US Federal Government officers or employees acting as part of their official duties; on a worldwide, perpetual, irrevocable, royalty-free basis to BMJ Publishing Group Ltd ("BMJ") its licensees and where the relevant Journal is co-owned by BMJ to the co-owners of the Journal, to publish the Work in this journal and any other BMJ products and to exploit all rights, as set out in our licence.

The Submitting Author accepts and understands that any supply made under these terms is made by BMJ to the Submitting Author unless you are acting as an employee on behalf of your employer or a postgraduate student of an affiliated institution which is paying any applicable article publishing charge ("APC") for Open Access articles. Where the Submitting Author wishes to make the Work available on an Open Access basis (and intends to pay the relevant APC), the terms of reuse of such Open Access shall be governed by a Creative Commons licence – details of these licences and which Creative Commons licence will apply to this Work are set out in our licence referred to above.

Other than as permitted in any relevant BMJ Author's Self Archiving Policies, I confirm this Work has not been accepted for publication elsewhere, is not being considered for publication elsewhere and does not duplicate material already published. I confirm all authors consent to publication of this Work and authorise the granting of this licence.

For peer review only

The Pharmacogenetics to Avoid Loss of Hearing (PALOH) Trial: A Protocol for a Prospective Observational Implementation Trial

McDermott JH[1,2†], Mahood R [1,2], Stoddard D[3], Mahaveer A[4], Turner M[5], Corry R[1], Garlick J[1], Miele G[6], Ainsworth S[6], Kemp L[6], Bruce IA[7,8] Body R[9,10], Ulph F[11], MacLeod R[1], Harvey K[5], Booth N[4], Roberts P[12] Wilson P[13], Newman WG[1,2†]

1. Manchester Centre for Genomic Medicine, St Mary's Hospital, Manchester University NHS Foundation Trust, Manchester, M13 9WL, UK
2. Division of Evolution and Genomic Sciences, School of Biological Sciences, University of Manchester, Manchester, UK
3. DS Analytics and Machine Learning Ltd, 12 Hammersmith Grove, Hammersmith, London, M6 7AP
4. Newborn Intensive Care Unit, Manchester University NHS Foundation Trust, Manchester, M13 9WL, UK
5. Newborn Intensive Care Unit, Liverpool Women's Hospital, Liverpool Crown St, Liverpool L8 7SS, UK
6. Genedrive Diagnostics Ltd, 48 Grafton Street, Manchester, M13 9XX
7. Hearing Health Theme Manchester NIHR Biomedical Research Centre, Manchester, UK
8. Pediatric ENT Department, Royal Manchester Children's Hospital, Manchester University NHS Foundation Trust, Manchester, United Kingdom, M13 9WL, UK
9. Emergency Department, Manchester University NHS Foundation Trust, Manchester, M13 9WL, UK
10. Division of Cardiovascular Sciences, University of Manchester, Manchester, UK
11. Division of Psychology & Mental Health, School of Health Sciences, Faculty of Biology, Medicine and Health, University of Manchester, Manchester Academic Health Science Centre, Oxford Road, Manchester M13 9PL, UK
12. Market Access & Reimbursement Solutions Ltd, 43 Castle Street, Liverpool, Merseyside
13. Alliance Manchester Business School, University of Manchester, Manchester, UK

†Correspondence to Professor William Newman & Dr John Henry McDermott, Manchester Centre for Genomic Medicine, Manchester University Hospitals NHS Foundation Trust, Manchester M13 9WL, UK

Tel: 0161 276 6506
e-mail: william.newman@manchester.ac.uk & john.mcdermott2@mft.nhs.uk

Running Title: Point of care genetic testing to prevent hearing loss

Word Count (Including References): 3893

Keywords: Pharmacogenetics, point-of-care, gentamicin, hearing-loss, neonates, genetics

For peer review only - http://bmjopen.bmj.com/site/about/guidelines.xhtml

Abstract

Introduction: In conjunction with a beta-lactam, aminoglycosides are the first-choice antibiotic for empirical treatment of sepsis in the neonatal period. The m.1555A>G variant predisposes to ototoxicity after aminoglycoside administration and has a prevalence of 1 in 500. Current genetic testing can take over 24-hours, an unacceptable delay in the acute setting. This prospective-observational trial will implement a rapid point of care test (POCT), facilitating tailored antibiotic prescribing to avoid hearing loss.

Methods: The genedrive™ POCT can detect the m.1555A>G variant in 26 minutes from buccal swab. This system will be integrated into the clinical pathways at two large UK neonatal centres over a minimum 6-month period. The primary outcome is the number of neonates successfully tested for the variant out of all babies prescribed antibiotics. As a secondary outcome, clinical timings will be compared to data collected prior to implementation, measuring the impact on routine practice.

Ethics and Dissemination: An opt-out consenting process was approved by the Research Ethics Committee (REC) in August 2019. Results will be published in full on completion of the study.

Discussion: The m.1555A>G variant is associated with aminoglycoside induced ototoxicity. This was established in 1993, yet technology has not been available to rapidly test for the variant. The genedrive™ assay can detect the variant in a practice-changing timeframe. However, it has not been established whether such a test could be practically implemented. To our knowledge, this trial represents the first example of a genetic POCT being employed in the neonatal setting to alter management acutely.

For peer review only - http://bmjopen.bmj.com/site/about/guidelines.xhtml

Strengths and Limitations

- This manuscript describes a prospective observational implementation trial of a rapid genetic Point of Care Test (POCT) in the acute neonatal setting to tailor antibiotic therapy, aiming to reduce aminoglycoside induced hearing loss.

- We embed a genetic POCT within the admission pathways of two Neonatal Intensive Care Units and, due to time pressure, prospective consenting is not possible, requiring the implementation of a unique ethical framework described within the protocol.

- To our knowledge this is the first example of a genetic POCT implemented in the acute neonatal setting to alter management.

- Rather than assessing the characteristics or performance of the assay itself during the trial, the primary and secondary outcomes focused on the utilization of the system and whether normal clinical practice was impacted following implementation.

- A limitation is that, given the relatively rare nature of the variant (1 in 500), it may transpire that the variant is never detected throughout the study, meaning some secondary outcomes cannot be reliably assessed.

For peer review only

**Title**	Pharmacogenetics to Avoid Loss of Hearing (PALOH): Study Protocol for a Prospective Observational Implementation Trial
**Trial registration**	ISRCTN13704894
**Protocol version**	V 1.3
**Funding**	NIHR Invention for Innovation (i4i) Programme. Grant Number II-LB-0417-20002
**Contact information for the trial sponsor**	Manchester University NHS Foundation Trust<br>Research Office<br>Manchester University NHS Foundation Trust<br>Oxford Road<br>Manchester<br>M13 9PL<br>Tel: 01612764125<br>Fax: 01612765766
**Role of sponsor**	The Sponsor, Manchester University NHS Foundation Trust, takes overall responsibility for proportionate, effective arrangements being in place to set up, run and report this research project. Sponsorship responsibilities include; ensuring suitability of the research proposal and researchers involved, facilitating peer review, promoting research transparency, ensuring that the necessary regulatory approvals are in place prior to study start, that there is adequate funding for the duration of the research and that effective arrangements are in place for reporting and monitoring the research. The Trust aims to support MFT staff to initiate and undertake research with our patients and staff.

For peer review only

1. Background

Aminoglycosides are broad-spectrum antibiotics which act by binding to the 16S rRNA component of the bacterial 30S ribosomal subunit, resulting in the translation of truncated proteins.[1,2] These abnormal proteins stimulate a stress response within the bacteria culminating in cell death. Due to their low cost and effectiveness, they are one of the most frequently prescribed medicines globally.[3] The National Institute for Health and Care Excellence (NICE) advises the use of intravenous benzylpenicillin with gentamicin as the first-choice antibiotic regimen for empirical treatment of infection in the neonatal period.[4] This combination has the major advantage of having a narrow spectrum of activity and lower risk of antibiotic resistance compared to alternative antibiotic regimens, such as third generation cephalosporins which are recommended as second line agents.

The side effect profile from protracted courses of aminoglycosides is well known, with nephrotoxicity and ototoxicity commonly recognised in cohorts who receive large amounts of aminoglycoside.[5] An ototoxic compound is one that can cause damage to the auditory system (cochleotoxicity) or, less commonly, vestibular system (vestibulotoxicity). Careful analysis revealed that aminoglycoside-induced ototoxicity (AIO) clusters within families and this trait seemed to be transmitted maternally, suggesting mitochondrial inheritance. Further work demonstrated that this susceptibility was caused by a variant in the 12S rRNA (RNR1) m.1555A>G.[6] This causes a change in the conformation of the 12S rRNA, producing a structure more like the bacterial 16S rRNA, meaning aminoglycosides can more readily bind causing cellular toxicity. The impact of this aberrant binding is most apparent in the inner ear hair cells, leading to ototoxicity.

The m.1555A>G variant is strongly associated with AIO and has a reported prevalence of 0.2% (~1 in 500).[7] It has previously been suggested that genetic testing should be used in children requiring aminoglycosides to prevent hearing loss and that this approach would be cost-effective when balanced against the costs of lifelong deafness.[3,7] In many centers, children with cystic fibrosis are tested for the variant at diagnosis as it is expected that these individuals will require aminoglycoside

For peer review only

antibiotics at some stage in their lives. This is typically undertaken by a validated genotyping assay in an accredited diagnostic laboratory, a strategy which takes approximately 3-4 days to return a result. Understandably, this testing strategy is not suitable for use in clinical settings where administration of antibiotics is required within an hour of the decision to start treatment with antibiotics.

Currently there is an inability to test for the m.1555A>G variant in the acute setting such as babies admitted to the neonatal intensive care unit (NICU), representing approximately 90,000 admissions per year in the UK alone. By introducing a simple genetic point of care test (POCT) with a result in under 30 minutes, we have the potential to avoid approximately 180 cases of severe/profound irreversible deafness every year. Those babies with the m.1555A>G variant could be prescribed an equally effective second line antibiotic, such as a cephalosporin. The UK NICE guidance for early onset neonatal infection recommends a combination of gentamicin and benzylpenicillin as first-line therapy not due to superiority over other agents on an individual patient level, but because that regimen has a narrower spectrum of activity and therefore does not readily contribute towards the development of resistant bacterial pathogens.[4]

Alongside an industry partner, genedrive™, we have a developed a small and robust thermocycler platform for rapid point-of-care diagnostic testing of the m.1555A>G variant which has been CE certified for clinical use. This protocol describes a multi-centre prospective observational trial to assess the implementation of the POCT in the clinical setting.

2. Methods

2.1 Study Design

Investigator-initiated, multi-centre, prospective-observational, implementation trial. Two study sites, both of which are recruiting and experimental.

2.2 Primary Objective

For peer review only

For peer review only - http://bmjopen.bmj.com/site/about/guidelines.xhtml

To critically assess the performance of a newly developed genetic POCT for use on the NICU and measure whether the clinical teams can integrate the test into their clinical practice without disrupting normal standard of care, facilitating tailored antibiotic prescribing.

2.3 Secondary Objectives

Exploratory objectives include an assessment of the wider impact of the implementation, including impact on resources and clinical timelines. We will also assess instances where the assay was not used and consider reasons for deviation. The reliability of the assay itself will also be measured through regular monitoring of results, assessment of test failures and confirmation against gold-standard genotyping.

2.4 Study Centres and Time Schedule

Participants will be recruited from two large UK based NICUs in the UK, Manchester University NHS Foundation Trust (MFT) and Liverpool Women's NHS Foundation Trust (LWH). Both sites will recruit participants and conduct the trial. It should be noted that there is considerable variation in antimicrobial prescribing practices both between and within different countries. The type of antibiotics used, dosing regimens, and monitoring protocols can vary between different nations, cities, and institutions.[8] Both units in this study use a combination of a beta-lactam and an aminoglycoside as first line therapy for early onset neonatal infection. Based on historical data, the majority of admissions to both units will be screened for infection and, as such, considered for antibiotic therapy. The trial began in January 2020 and the last participant was recruited in November 2020. The study continued despite the SARS-CoV-2 pandemic.

2.5 Primary Outcome

The primary outcome is the number of neonates who are successfully tested for the m.1555A>G variant via this novel POCT genetic test as a proportion of all babies who receive aminoglycoside antibiotics on the two participating NICUs.

For peer review only - http://bmjopen.bmj.com/site/about/guidelines.xhtml

2.6 Secondary Outcomes

This trial will also assess several secondary outcomes including the total number of neonates identified with the m.1555A>G genetic variant (positive result) as a proportion of the study population. An assessment of resource impact will be undertaken, including a health economic analysis of additional staff time to secure sample and testing and costs associated with alternative antibiotic prescribing for any positive tests. We will record the number of neonates where testing was not undertaken and the rationale for this. Correlation of the POCT result with the current in-house reference assay will be assessed and any test fails will be reported. In the acute neonatal setting, delivering antibiotics in a timely manner is critical and this should ideally be within an hour after admission. As such, we will assess whether the time to antibiotic administration after admission is impacted by implementation of the genetic POCT. A reference time to antibiotic was measured over a month-long period before implementation.

2.7 Inclusion criteria

All babies admitted to NICU across the two participating sites, commencing from the trial start date. This includes babies admitted directly from delivery suite, midwifery led units or transferred from another neonatal unit. At the LWH site, babies who are screened for infection on the NICU but then transferred to external wards (not formally admitted to NICU) will also be included. It is a clinical decision whether the babies are being screened for infection as part of their assessment, reflecting the real-world, pragmatic, nature of the trial. This also reflects the variation in admission procedures between the two trial sites where pathways differ due to local practices.

Given that all babies admitted to NICU are eligible for recruitment, some of those recruited will not go onto receive antibiotic therapy. If the baby did not go onto receive antibiotics, their data will still form part of the dataset for analysis.

2.8 Exclusion Criteria

For peer review only - http://bmjopen.bmj.com/site/about/guidelines.xhtml

Babies requiring antibiotics immediately, as determined by the admitting clinician, with already established intravenous (IV) access. These exclusion criteria recognize the potential urgency of any decision to deliver antibiotics. If IV access has not been achieved, then this may provide time to run the assay. We expect that only a small proportion of admissions would be excluded based on these criteria.

2.9 Training

Training of a minimum of 80% of relevant nursing and medical staff within the two NICUs will commence in the 6 months prior to study start. This figure has been chosen, based on guidance from senior NICU clinicians and nursing staff, to ensure that there will always be staff members on-shift who are trained to undertake the assay throughout the study.

Training will include practical use and interpretation of the assay, with Standard Operating Procedures for use integrated into the standard admission procedure. A “train the trainer” approach will be adopted, where a number of experienced NICU research nurses plus additional clinical nursing staff identified as “super-users” will receive training directly from representatives of the device manufacturer. These designated super-users will then cascade training to the remaining nursing and medical staff within the two neonatal services.

Ongoing refresher training and online resources will be made available throughout the trial. A training log will be maintained detailing staff competency including dates of training, in order to identify any correlation of assay performance with increasing training numbers. NICU research nurses and co-investigators will be fully Good Clinical Practice (GCP) competent. A local, named co-investigator (neonatal consultant) will be responsible for clinical staff performing any duties in accordance with the protocol, GCP and local requirements.

2.10 Testing Process & Assessment

For peer review only - http://bmjopen.bmj.com/site/about/guidelines.xhtml

Participants will be recruited as they are admitted or reviewed by the NICU team. Given the acute nature of the project and the timeframes involved, there is no opportunity to undertake a formal pre-trial screening assessment or prospective consenting process. All babies admitted to NICU or screened for infection by the NICU team will be deemed eligible for recruitment unless there is a clinical decision by the responsible admitting clinician that the exclusion criterion is met and the individual requires immediate antibiotic therapy, without delay.

Following recruitment, the time that a decision was made to prescribe antibiotics represents time 0 (Figure 1). If antibiotics were not prescribed, then time 0 is represented by the time of admission. The genetic POCT is performed from a buccal swab. When babies are admitted to NICU several swabs are already taken at admission. Where babies are recruited to the study, the m.1555A>G buccal swab will be performed alongside these normal admission processes. The assay will be commenced, and normal practice will continue. Once the assay result is available, it can be used to then undertake personalized antibiotic prescribing, avoiding aminoglycoside antibiotics if the m.1555A>G variant is detected. The CE certified assay will be integrated into the admission pathways at both centres and clinicians will not be blinded to the results.

As part of the study the following variables will be recorded; participant demographics, admission time, time of decision to prescribe, time swab taken, time assay started, time of assay result, assay result, time of antibiotic prescription, antibiotics prescribed. Where a positive result is identified and antibiotic therapy is required, an alternative antibiotic regimen (standard non-gentamicin pathway as per hospital guidelines) will be prescribed and a referral should be made to clinical genetics for familial cascade testing. Where there is a test-fail, standard of care should be followed, i.e the baby should be administered gentamicin if clinically indicated. Negative results will not be formally reported to the parents. However, all parents will be provided with information on the study in their information pack, allowing them to contact the study team for further information if they wish.

For peer review only - http://bmjopen.bmj.com/site/about/guidelines.xhtml

Assay performance will be assessed throughout the study, with at least 10% of samples each month tested via gold-standard sequencing. Any positive samples will also be confirmed as per standard clinical pathways. Any divergence between the POCT and gold-standard methodology will be reviewed by the trial management committee.

Data collection will be undertaken contemporaneously by the clinical staff and any missing data will be collected by the NICU research nurses. Data will be entered into an electronic database by the clinical team and a study ID will be generated. The participants will subsequently be anonymized to the research team. If an individual is not tested for any reason (e.g. test overlooked, staff availability or the individual meets the exclusion criteria), these incidences will be captured retrospectively by NICU research nurses by checking daily admission records and the reasons will be documented.

2.11 Samples Size

The sample size (approximately 900 babies) is estimated based upon the expected number of admissions across both clinical sites over an approximate 10-month trial period. To achieve a statistical power of 90% with a 5% significance level, a margin of 0.5 (in Cohen's d, which equates to about 7 minutes difference given the standard deviation in the control sample) and a control sample of N = 50, a test sample size of 112 is required. With the forecast test sample size of 900, statistical power should be around 95% to detect variance in clinical timings before and after implementation.

2.12 Statistical Analysis

For the primary outcome, simple descriptive statistics will be used to provide a metric for the proportion of babies tested for the variant over the number who were ultimately prescribed aminoglycoside antibiotics.

Regarding the secondary outcome measures, the objective is to establish that the introduction of the POCT does not significantly impact the time between admission to NICU and the prescription of antibiotics for acute admissions. The burden of proof rests on the research hypothesis, meaning the

For peer review only - http://bmjopen.bmj.com/site/about/guidelines.xhtml

null hypothesis is that the new POCT does impact this quantity. This is a so-called non-inferiority significance test. The quantity of interest is the mean difference between the admission to ICU and prescription of antibiotics timestamps in two situations: with and without the POCT test $(\mu_c, \mu_{t)})$. The null hypothesis is that the difference between these quantities is greater than or equal to some prespecified margin (Δ) i.e. $H_0{:}\mu_c - \mu_t \geq \Delta; H_A{:}U_t - U_c < \Delta$. A type I error here is concluding the time durations are equivalent when in fact the time to antibiotic with the POCT is greater.

The two samples are of unequal size, with the control ('before implementation') sample having about n_c=50, and a forecasted test sample size of around n_t=900. We also have no insight into population variance and cannot assume they are the same in both groups (it may for example take some staff members longer to carry out the new test than others). As such, a one-sided Welch t-test will be used to reject or not reject the null hypothesis.

Throughout the study we anticipate that there will be iterative updates to the POCT technology, as avenues for optimization emerge because of our findings from real-world implementation. The timings of these updates will be recorded and separate post-hoc statistical analysis will be undertaken to assess the impact of each update.

2.13 Study Organisation

Two groups have been convened to oversee the trial, a management group, and a steering committee. The Trial Management Group convenes monthly to ensure all practical details of the trial are progressing well, within the agreed milestones, and is led by the study Chief Investigator. The trial management group will act as the data monitoring committee. Membership is independent of the sponsors. The data management team will have access to anonymized data and will review assay performance and trial conduct on a monthly basis- specifically the concordance with gold standard. All data will be analyzed by an independent statistical team who report monthly to the trial

For peer review only - http://bmjopen.bmj.com/site/about/guidelines.xhtml

management group. Any amendments to the protocol will be communicated to the REC, funding agency and the trials registry.

The Trial Steering Committee meets on a 6 monthly basis to monitor the performance of the trial against the agreed project plan and advise on scientific and technical aspects of the project. The membership is independent of the Sponsor and Investigators.

A Stakeholders group has also been assembled to advise on the design and dissemination of the study intervention in the interests of the intended end users and participants. This membership includes personnel from key clinical services (neonatology and paediatric audiology), commissioners (NHS England) and newborn and hearing loss charities.

2.14 Patient and Public Involvement

Involvement of parent and public representatives has been a critical component of the development of this trial protocol. Parent representatives are involved in both the trial management groups, stakeholders committee and a separate Public and Patient Involvement and Engagement (PPIE) panel, providing both neonatal care and/or hearing loss experience. The PPIE panel was involved in the development of both the protocol and ethics application. The PI and co-investigators presented early versions of the texts to the PPIE panel and any potentially contentious issues were discussed in more detail. The insights gained from these meetings were used to refine the applications prior to submission.

2.15 Ethics and Adverse Events

This is the world's first trial, that we are aware of, of a genetic POCT to personalize medicine in the acute neonatal setting. As such, there have been several ethical challenges, mainly performing DNA analysis without qualifying consent. Prospective consent is not possible given the acute nature of the admission, therefore we sought permission to proceed with the POCT included as part of the standard of care. The parents could then opt-out of the data being used as part of the study. This ethical process

For peer review only - http://bmjopen.bmj.com/site/about/guidelines.xhtml

has been described in detail previously.[9] Approval was provided by the Research Ethics Committee (REC) and the Human Research Authority (HRA) in August 2019. We will record any adverse event related to the sample collection procedure or directly related to the testing process.

2.16 Dissemination of Results

The results of the study will be submitted to international peer-reviewed scientific journals, irrespective of their outcome and presented as per best practice STROBE guidelines for observational trials.

3. Discussion

To our knowledge, the PALOH study represents the first trial of a genetic POCT designed to alter management in an acute, time-sensitive setting. Successful implementation could avoid up to 180 cases of aminoglycoside induced ototoxicity in the UK each year, and many more worldwide. However, this study has implications beyond this gene-drug pair. The anticipated results will indicate whether genetic technology can be implemented in the acute setting more broadly, a hitherto untested concept. The unprecedented nature of this work led to several practical and ethical challenges in the design of the protocol.

Several studies have previously reported that most clinicians are not confident with the interpretation and implementation of genetic data.[10–12] With this in mind, we ensured that the assay implemented as part of this trial was as straightforward to interpret as possible, providing a binary readout for the presence or absence of the m.1555A>G variant. However, even if an assay is simple to operate and interpret, we recognized that the imposition of any alien process in such a critical time-period could impact adoption. We therefore designed this protocol to include a comprehensive training strategy prior to the trial commencing on both units. This significant undertaking ensures that over 500 health care professionals, greater than 80% of all staff, have exposure to the system before implementation.

Due to CE certification requirements for medical devices in the European Union (EU), any new system must be validated prior to clinical implementation. For a technology which has been designed for use

For peer review only - http://bmjopen.bmj.com/site/about/guidelines.xhtml

For peer review only

in the acute setting, this requirement poses unique challenges. Although we were able to thoroughly assess the technology in the pre-clinical laboratory setting, satisfying the requirements for CE certification, it was not possible to perform analytical validation in its intended use setting, the NICU. This was because any result generated in this setting would be actionable and could impact management, precipitating an ethical dilemma. As a result, we determined that the most appropriate strategy was to regularly review the performance of the assay following implementation and introduce any updates to the training or technology as required. We have therefore made clear in our analysis plan that outcome statistics will be reported for the study as a whole and for each time-period post-update, if any such updates are necessary.

Given the acute context in which the PALOH trial is taking place, it was recognised that it would not be practicable for informed consent to be gained prospectively. Newborns admitted to NICU are, by definition, acutely unwell and it is likely to represent one of the most stressful time in a parent's life.

As such, it was determined that asking for consent to perform this genetic test immediately after birth would be inappropriate. Following discussion with stakeholders including parents, neonatologists, and geneticists, ethical approval was sought based on the POCT being included as part of the standard package of care offered to the acutely unwell neonate, for which broad parental consent is typically provided, or if unavailable, is undertaken in the best interest of the child.[13] Our PPIE panel were particularly helpful during this process, providing a forum to discuss the various consenting options where we could consider relative benefits and costs of each strategy.

Neonates are tested for the variant as part of their admission process via a buccal swab, the variant details then used to inform prescribing and data is subsequently gathered and analysed. Parents will be provided information regarding the trial by the NICU team as part of an existing parental information pack. They would have the right to ask for their child's data to be removed from the study at that stage. This provides a mechanism to opt-out prior to the analysis stage although, critically, by

For peer review only - http://bmjopen.bmj.com/site/about/guidelines.xhtml

this point their child will have already had tailored antibiotic prescribing based on the presence or absence of the variant.

To our knowledge this represents the first example of such a consenting model being used in a trial involving genetic testing. In the summer of 2019, an application for ethical approval was submitted to the Health Research Authority Research Ethics Service (RES), proposing that the importance of this genetic test and the potential benefits warranted approval of an opt-out consenting model. After consideration, the REC approved the PALOH trial design in full pending HRA approval, making it the first trial of a genetic POCT to alter management in the acute setting. However, in the week following the REC meeting the PALOH study team received notice that the HRA were withholding approval as they had concerns that the study was in violation of the Human Tissue Act (2004) (HT Act). Specifically, the HRA felt that the trial design did not meet the legal definition of "qualifying consent" as outlined in the HT Act.

Following protracted ethical and legal discussion, the trial design was approved following the agreement that the act of performing the initial test represents a clinical decision, rather than a research question. This approach was then permissible under Schedule 4 Part 2 of the HT Act which outlines that "the medical diagnosis or treatment of the person whose body manufactured the DNA" is an excepted purpose for DNA analysis without formal consent.[9] We believe that the PALOH study represents the first time this ethicolegal issue has been considered. As such, our methodology may be of value to future clinical academics exploring the application of similar pharmacogenetic biomarkers.

The anticipated results of the PALOH trial will assess whether a genetic POCT can be used to tailor antibiotic prescribing in the acute NICU setting. This will be of value not only in avoiding AIO but also in optimising management in other disease areas, as the relevance of genetics in everyday clinical practice continues to develop. We believe that this trial methodology and the ethical issues outlined in this work will be of value when researchers are designing similar studies in the future.

For peer review only - http://bmjopen.bmj.com/site/about/guidelines.xhtml

4. Trial Status

The PALOH Study has closed to recruitment and analysis is ongoing. Recruitment began in January 2020 and completed in November 2020.

5. Declarations

Ethics Approval: Health Research Authority (HRA) and Health and Care Research Wales (HCRW) approval was granted by the Research Ethics Committee at Liverpool Women's Hospital on the 23rd of September 2019. REC Reference 19/NW/0400.

Consent for Publication: Consent for publication is not applicable as there are no identifying images or other personal details of participants presented in this protocol manuscript.

Competing Interests: SA, LK and GM are employees of genedrive. The other authors report no competing interests.

Data: All results of this trial will be submitted for peer reviewed publication. Final data will be submitted to an open access repository on publication of the final trial manuscript.

Author Statements: JHM and WGN developed the concept for the study. SA, LK and GM designed and optimized the assay for implementation. All authors contributed to the development of the protocol led by AM and MT and the Manchester Foundation Trust and Liverpool Women's Hospital sites, respectively. KH and NB manage the data collection at each clinical site. RM is research manager for PALOH. DS, PR, and PW are involved in data analysis. RMc and FU have designed and undertaken stakeholder interviews which contribute to the design of the protocol. JG and RC, along with RM, have overseen the PPIE process. RB, IB and PW have advised on the implementation process and broader study design. All authors are responsible for either the recruitment of participants, data collection or data analysis. All authors read and approved the final version of this manuscript.

Acknowledgements: The authors would like to thank the patients and family members who took part in the study. We would like to acknowledge the huge efforts of the nurses, physicians and administrative staff at both study sites who have been instrumental in developing the project. We are very grateful to the parents and public representatives who sat on the PPIE committee and advised on the development of the project.

For peer review only

Funding: Funding for the project was provided via an NIHR i4i program award (Grant Number: II-LB-0417-20002), with additional support from the Manchester NIHR Biomedical Research Centre, Hearing Health Theme (IS-BRC-1215-20007). Pilot data support from Action on Hearing Loss Flexi grant F67_Newm.

Figure Legends

Figure 1. Flow diagram demonstrating the admission, testing, and prescribing process in the PALOH Trial. The time that a decision was made to prescribe antibiotics represents time 0. If antibiotics were not prescribed, then time 0 is represented by the time of admission.

For peer review only

References

1 Schacht J, Talaska AE, Rybak LP. Cisplatin and Aminoglycoside Antibiotics: Hearing Loss and Its Prevention. *Anat Rec (Hoboken)* 2012; **295**: 1837–1850.

2 Lanvers-Kaminsky C, Ciarimboli G. Pharmacogenetics of drug-induced ototoxicity caused by aminoglycosides and cisplatin. *Pharmacogenomics* 2017; **18**: 1683–1695.

3 Bitner-Glindzicz M, Rahman S. Ototoxicity caused by aminoglycosides. *BMJ* 2007; **335**: 784–785.

4 National Institute for Health and Care Excellence. CG149. Neonatal infection (early onset): antibiotics for prevention and treatment. 2012.

5 Prayle A, Watson A, Fortnum H, Smyth A. Side effects of aminoglycosides on the kidney, ear and balance in cystic fibrosis. *Thorax* 2010; **65**: 654–658.

6 Prezant TR, Agapian JV, Bohlman MC, Bu X, Öztas S, Qiu W-Q *et al.* Mitochondrial ribosomal RNA mutation associated with both antibiotic–induced and non–syndromic deafness. *Nature Genetics* 1993; **4**: 289–294.

7 Bitner-Glindzicz M, Pembrey M, Duncan A, Heron J, Ring SM, Hall A *et al.* Prevalence of Mitochondrial 1555A→G Mutation in European Children. *N Engl J Med* 2009; **360**: 640–642.

8 Gkentzi D, Dimitriou G. Antimicrobial Stewardship in the Neonatal Intensive Care Unit: An Update. *Curr Pediatr Rev* 2019; **15**: 47–52.

9 McDermott JH. Genetic testing in the acute setting: a round table discussion. *Journal of Medical Ethics* 2020; **46**: 531–532.

10 Christensen KD, Vassy JL, Jamal L, Lehmann LS, Slashinski MJ, Perry DL *et al.* Are physicians prepared for whole genome sequencing? a qualitative analysis. *Clin Genet* 2016; **89**: 228–234.

11 Hauser D, Obeng AO, Fei K, Ramos MA, Horowitz CR. Views Of Primary Care Providers On Testing Patients For Genetic Risks For Common Chronic Diseases. *Health Aff (Millwood)* 2018; **37**: 793–800.

For peer review only - http://bmjopen.bmj.com/site/about/guidelines.xhtml

12 Selkirk CG, Weissman SM, Anderson A, Hulick PJ. Physicians' preparedness for integration of genomic and pharmacogenetic testing into practice within a major healthcare system. *Genet Test Mol Biomarkers* 2013; **17**: 219–225.

13 Coulson-Smith P, Lucassen A. Using biomarkers in acute medicine to prevent hearing loss: should this require specific consent? *Journal of Medical Ethics* 2020; **46**: 536–537.

For peer review only

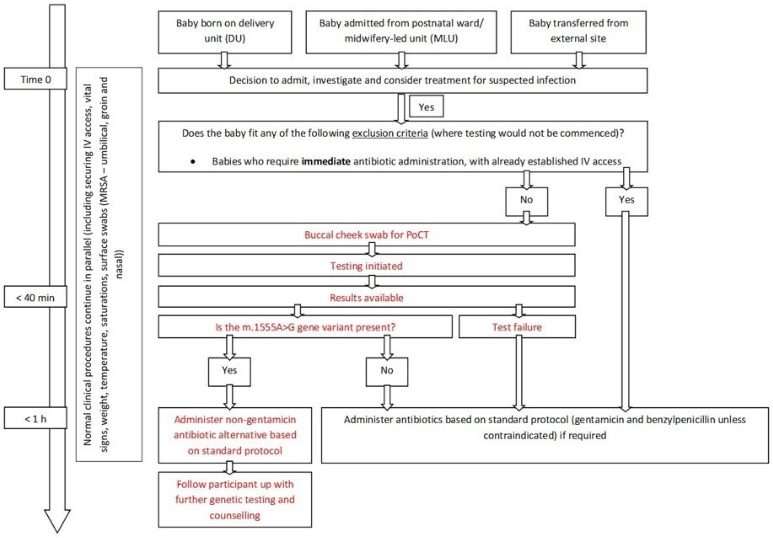

Figure 1. Flow diagram demonstrating the admission, testing, and prescribing process in the PALOH Trial. The time that a decision was made to prescribe antibiotics represents time 0. If antibiotics were not prescribed, then time 0 is represented by the time of admission.

100x60mm (300 x 300 DPI)

STROBE Statement—checklist of items that should be included in reports of observational studies

	Item No	Recommendation	Page No
**Title and abstract**	1	(*a*) Indicate the study's design with a commonly used term in the title or the abstract	1
		(*b*) Provide in the abstract an informative and balanced summary of what was done and what was found	2
**Introduction**			
Background/rationale	2	Explain the scientific background and rationale for the investigation being reported	5
Objectives	3	State specific objectives, including any prespecified hypotheses	6
**Methods**			
Study design	4	Present key elements of study design early in the paper	6
Setting	5	Describe the setting, locations, and relevant dates, including periods of recruitment, exposure, follow-up, and data collection	7
Participants	6	(*a*) *Cohort study*—Give the eligibility criteria, and the sources and methods of selection of participants. Describe methods of follow-up<br>*Case-control study*—Give the eligibility criteria, and the sources and methods of case ascertainment and control selection. Give the rationale for the choice of cases and controls<br>*Cross-sectional study*—Give the eligibility criteria, and the sources and methods of selection of participants	7 & 8
		(*b*) *Cohort study*—For matched studies, give matching criteria and number of exposed and unexposed<br>*Case-control study*—For matched studies, give matching criteria and the number of controls per case	N/A
Variables	7	Clearly define all outcomes, exposures, predictors, potential confounders, and effect modifiers. Give diagnostic criteria, if applicable	7
Data sources/ measurement	8*	For each variable of interest, give sources of data and details of methods of assessment (measurement). Describe comparability of assessment methods if there is more than one group	*9*
Bias	9	Describe any efforts to address potential sources of bias	13
Study size	10	Explain how the study size was arrived at	9 & 10
Quantitative variables	11	Explain how quantitative variables were handled in the analyses. If applicable, describe which groupings were chosen and why	10
Statistical methods	12	(*a*) Describe all statistical methods, including those used to control for confounding	10
		(*b*) Describe any methods used to examine subgroups and interactions	N/A
		(*c*) Explain how missing data were addressed	N/A
		(*d*) *Cohort study*—If applicable, explain how loss to follow-up was addressed<br>*Case-control study*—If applicable, explain how matching of cases and controls was addressed<br>*Cross-sectional study*—If applicable, describe analytical methods taking account of sampling strategy	N/A
		(*e*) Describe any sensitivity analyses	N/A

For peer review only - http://bmjopen.bmj.com/site/about/guidelines.xhtml

**Results**			
Participants	13*	(a) Report numbers of individuals at each stage of study—eg numbers potentially eligible, examined for eligibility, confirmed eligible, included in the study, completing follow-up, and analysed	Awaited
		(b) Give reasons for non-participation at each stage	Awaited
		(c) Consider use of a flow diagram	Awaited
Descriptive data	14*	(a) Give characteristics of study participants (eg demographic, clinical, social) and information on exposures and potential confounders	Awaited
		(b) Indicate number of participants with missing data for each variable of interest	Awaited
		(c) *Cohort study*—Summarise follow-up time (eg, average and total amount)	Awaited
Outcome data	15*	*Cohort study*—Report numbers of outcome events or summary measures over time	Awaited
		Case-control study—Report numbers in each exposure category, or summary measures of exposure	Awaited
		Cross-sectional study—Report numbers of outcome events or summary measures	Awaited
Main results	16	(*a*) Give unadjusted estimates and, if applicable, confounder-adjusted estimates and their precision (eg, 95% confidence interval). Make clear which confounders were adjusted for and why they were included	Awaited
		(*b*) Report category boundaries when continuous variables were categorized	Awaited
		(*c*) If relevant, consider translating estimates of relative risk into absolute risk for a meaningful time period	Awaited
Other analyses	17	Report other analyses done—eg analyses of subgroups and interactions, and sensitivity analyses	Awaited
**Discussion**			
Key results	18	Summarise key results with reference to study objectives	Awaited
Limitations	19	Discuss limitations of the study, taking into account sources of potential bias or imprecision. Discuss both direction and magnitude of any potential bias	Awaited
Interpretation	20	Give a cautious overall interpretation of results considering objectives, limitations, multiplicity of analyses, results from similar studies, and other relevant evidence	Awaited
Generalisability	21	Discuss the generalisability (external validity) of the study results	Awaited
**Other information**			
Funding	22	Give the source of funding and the role of the funders for the present study and, if applicable, for the original study on which the present article is based	3

*Give information separately for cases and controls in case-control studies and, if applicable, for exposed and unexposed groups in cohort and cross-sectional studies.

Note: An Explanation and Elaboration article discusses each checklist item and gives methodological background and published examples of transparent reporting. The STROBE checklist is best used in conjunction with this article (freely available on the Web sites of PLoS Medicine at http://www.plosmedicine.org/, Annals of Internal Medicine at http://www.annals.org/, and Epidemiology at http://www.epidem.com/). Information on the STROBE Initiative is available at www.strobe-statement.org.

For peer review only

BMJ Open

The Pharmacogenetics to Avoid Loss of Hearing (PALOH) Trial: A Protocol for a Prospective Observational Implementation Trial

Journal:	*BMJ Open*
Manuscript ID	bmjopen-2020-044457.R2
Article Type:	Protocol
Date Submitted by the Author:	19-May-2021
Complete List of Authors:	McDermott, John; Manchester University NHS Foundation Trust, Manchester Centre for Genomic Medicine ; The University of Manchester, Division of Evolution and Genomic Sciences<br>Mahood, Rachel; Manchester University NHS Foundation Trust, Manchester Centre for Genomic Medicine<br>Stoddard, Duncan; DS Analytics and Machine Learning Ltd; Manchester University NHS Foundation Trust, Manchester Centre for Genomic Medicine<br>Mahaveer, Ajit; Manchester University NHS Foundation Trust, Neonatal Intensive Care Unit<br>Turner, Mark; Liverpool Women's Hospital NHS Foundation Trust<br>Corry, Rachel; Manchester University NHS Foundation Trust, Manchester Centre for Genomic Medicine<br>Garlick, Julia; Manchester University NHS Foundation Trust, Manchester Centre for Genomic Medicine<br>Miele, Gino; Genedrive plc<br>Ainsworth, Shaun; Genedrive plc<br>Kemp, Laura; Genedrive plc<br>Bruce, Iain; Manchester University Hospitals NHS Foundation Trust, Paediatric ENT Department<br>Body, Richard; Manchester University NHS Foundation Trust, Emergency Department; The University of Manchester, Division of Cardiovascular Sciences<br>Ulph, Fiona; University of Manchester<br>Macleod, Rhona; Manchester University NHS Foundation Trust, Manchester Centre for Genomic Medicine<br>Harvey, Karen; Liverpool Women's Hospital NHS Foundation Trust<br>Booth, Nicola; Manchester University NHS Foundation Trust, Neonatal Intensive Care Unit<br>Roberts, Peter; Market Access & Reimbursement Solutions<br>Wilson, Paul; University of Manchester, Alliance Manchester Business School<br>Newman, William; Manchester University NHS Foundation Trust, Manchester Centre for Genomic Medicine; University of Manchester, Division of Evolution and Genomic Sciences
**Primary Subject Heading**:	Genetics and genomics

For peer review only - http://bmjopen.bmj.com/site/about/guidelines.xhtml

Secondary Subject Heading:	Genetics and genomics, Paediatrics, Pharmacology and therapeutics
Keywords:	Neonatal intensive & critical care < INTENSIVE & CRITICAL CARE, GENETICS, Audiology < OTOLARYNGOLOGY, Adverse events < THERAPEUTICS, PREVENTIVE MEDICINE

SCHOLARONE™
Manuscripts

I, the Submitting Author has the right to grant and does grant on behalf of all authors of the Work (as defined in the below author licence), an exclusive licence and/or a non-exclusive licence for contributions from authors who are: i) UK Crown employees; ii) where BMJ has agreed a CC-BY licence shall apply, and/or iii) in accordance with the terms applicable for US Federal Government officers or employees acting as part of their official duties; on a worldwide, perpetual, irrevocable, royalty-free basis to BMJ Publishing Group Ltd ("BMJ") its licensees and where the relevant Journal is co-owned by BMJ to the co-owners of the Journal, to publish the Work in this journal and any other BMJ products and to exploit all rights, as set out in our licence.

The Submitting Author accepts and understands that any supply made under these terms is made by BMJ to the Submitting Author unless you are acting as an employee on behalf of your employer or a postgraduate student of an affiliated institution which is paying any applicable article publishing charge ("APC") for Open Access articles. Where the Submitting Author wishes to make the Work available on an Open Access basis (and intends to pay the relevant APC), the terms of reuse of such Open Access shall be governed by a Creative Commons licence – details of these licences and which Creative Commons licence will apply to this Work are set out in our licence referred to above.

Other than as permitted in any relevant BMJ Author's Self Archiving Policies, I confirm this Work has not been accepted for publication elsewhere, is not being considered for publication elsewhere and does not duplicate material already published. I confirm all authors consent to publication of this Work and authorise the granting of this licence.

For peer review only

The Pharmacogenetics to Avoid Loss of Hearing (PALOH) Trial: A Protocol for a Prospective Observational Implementation Trial

McDermott JH[1,2†], Mahood R [1,2], Stoddard D[3], Mahaveer A[4], Turner M[5], Corry R[1], Garlick J[1], Miele G[6], Ainsworth S[6], Kemp L[6], Bruce IA[7,8] Body R[9,10], Ulph F[11], MacLeod R[1], Harvey K[5], Booth N[4], Roberts P[12] Wilson P[13], Newman WG[1,2†]

1. Manchester Centre for Genomic Medicine, St Mary's Hospital, Manchester University NHS Foundation Trust, Manchester, M13 9WL, UK
2. Division of Evolution and Genomic Sciences, School of Biological Sciences, University of Manchester, Manchester, UK
3. DS Analytics and Machine Learning Ltd, 12 Hammersmith Grove, Hammersmith, London, M6 7AP
4. Newborn Intensive Care Unit, Manchester University NHS Foundation Trust, Manchester, M13 9WL, UK
5. Newborn Intensive Care Unit, Liverpool Women's Hospital, Liverpool Crown St, Liverpool L8 7SS, UK
6. Genedrive Diagnostics Ltd, 48 Grafton Street, Manchester, M13 9XX
7. Hearing Health Theme Manchester NIHR Biomedical Research Centre, Manchester, UK
8. Pediatric ENT Department, Royal Manchester Children's Hospital, Manchester University NHS Foundation Trust, Manchester, United Kingdom, M13 9WL, UK
9. Emergency Department, Manchester University NHS Foundation Trust, Manchester, M13 9WL, UK
10. Division of Cardiovascular Sciences, University of Manchester, Manchester, UK
11. Division of Psychology & Mental Health, School of Health Sciences, Faculty of Biology, Medicine and Health, University of Manchester, Manchester Academic Health Science Centre, Oxford Road, Manchester M13 9PL, UK
12. Market Access & Reimbursement Solutions Ltd, 43 Castle Street, Liverpool, Merseyside
13. Alliance Manchester Business School, University of Manchester, Manchester, UK

†Correspondence to Professor William Newman & Dr John Henry McDermott, Manchester Centre for Genomic Medicine, Manchester University Hospitals NHS Foundation Trust, Manchester M13 9WL, UK

Tel: 0161 276 6506
e-mail: william.newman@manchester.ac.uk & john.mcdermott2@mft.nhs.uk

Running Title: Point of care genetic testing to prevent hearing loss

Word Count (Including References): 3893

Keywords: Pharmacogenetics, point-of-care, gentamicin, hearing-loss, neonates, genetics

For peer review only

Abstract

Introduction: In conjunction with a beta-lactam, aminoglycosides are the first-choice antibiotic for empirical treatment of sepsis in the neonatal period. The m.1555A>G variant predisposes to ototoxicity after aminoglycoside administration and has a prevalence of 1 in 500. Current genetic testing can take over 24-hours, an unacceptable delay in the acute setting. This prospective-observational trial will implement a rapid point of care test (POCT), facilitating tailored antibiotic prescribing to avoid hearing loss.

Methods and Analysis: The genedrive™ POCT can detect the m.1555A>G variant in 26 minutes from buccal swab. This system will be integrated into the clinical pathways at two large UK neonatal centres over a minimum 6-month period. The primary outcome is the number of neonates successfully tested for the variant out of all babies prescribed antibiotics. As a secondary outcome, clinical timings will be compared to data collected prior to implementation, measuring the impact on routine practice.

Ethics and Dissemination: Approval for the trial was granted by the Research Ethics Committee (REC) and Human Research Authority in August 2019. Results will be published in full on completion of the study.

For peer review only - http://bmjopen.bmj.com/site/about/guidelines.xhtml

Strengths and Limitations

- This manuscript describes a prospective observational implementation trial of a rapid genetic Point of Care Test (POCT) in the acute neonatal setting to tailor antibiotic therapy, aiming to reduce aminoglycoside induced hearing loss.
- We embed a genetic POCT within the admission pathways of two Neonatal Intensive Care Units and, due to time pressure, prospective consenting is not possible, requiring the implementation of a unique ethical framework described within the protocol.
- To our knowledge this is the first example of a genetic POCT implemented in the acute neonatal setting to alter management.
- Rather than assessing the characteristics or performance of the assay itself during the trial, the primary and secondary outcomes focused on the utilization of the system and whether normal clinical practice was impacted following implementation.
- A limitation is that, given the relatively rare nature of the variant (1 in 500), it may transpire that the variant is never detected throughout the study, meaning some secondary outcomes cannot be reliably assessed.

For peer review only

**Title**	The Pharmacogenetics to Avoid Loss of Hearing (PALOH) Trial: A Protocol for a Prospective Observational Implementation Trial
**Trial registration**	ISRCTN13704894
**Protocol version**	V 1.3
**Funding**	NIHR Invention for Innovation (i4i) Programme. Grant Number II-LB-0417-20002
**Contact information for the trial sponsor**	Manchester University NHS Foundation Trust<br>Research Office<br>Manchester University NHS Foundation Trust<br>Oxford Road<br>Manchester<br>M13 9PL<br>Tel: 01612764125<br>Fax: 01612765766
**Role of sponsor**	The Sponsor, Manchester University NHS Foundation Trust, takes overall responsibility for proportionate, effective arrangements being in place to set up, run and report this research project. Sponsorship responsibilities include; ensuring suitability of the research proposal and researchers involved, facilitating peer review, promoting research transparency, ensuring that the necessary regulatory approvals are in place prior to study start, that there is adequate funding for the duration of the research and that effective arrangements are in place for reporting and monitoring the research. The Trust aims to support MFT staff to initiate and undertake research with our patients and staff.

For peer review only

1. Background

Aminoglycosides are broad-spectrum antibiotics which act by binding to the 16S rRNA component of the bacterial 30S ribosomal subunit, resulting in the translation of truncated proteins.[1,2] These abnormal proteins stimulate a stress response within the bacteria culminating in cell death. Due to their low cost and effectiveness, they are one of the most frequently prescribed medicines globally.[3] The National Institute for Health and Care Excellence (NICE) advises the use of intravenous benzylpenicillin with gentamicin as the first-choice antibiotic regimen for empirical treatment of infection in the neonatal period.[4] This combination has the major advantage of having a narrow spectrum of activity and lower risk of antibiotic resistance compared to alternative antibiotic regimens, such as third generation cephalosporins which are recommended as second line agents.

The side effect profile from protracted courses of aminoglycosides is well known, with nephrotoxicity and ototoxicity commonly recognised in cohorts who receive large amounts of aminoglycoside.[5] An ototoxic compound is one that can cause damage to the auditory system (cochleotoxicity) or, less commonly, vestibular system (vestibulotoxicity). Careful analysis revealed that aminoglycoside-induced ototoxicity (AIO) clusters within families and this trait seemed to be transmitted maternally, suggesting mitochondrial inheritance. Further work demonstrated that this susceptibility was caused by a variant in the 12S rRNA (RNR1) m.1555A>G.[6] This causes a change in the conformation of the 12S rRNA, producing a structure more like the bacterial 16S rRNA, meaning aminoglycosides can more readily bind causing cellular toxicity. The impact of this aberrant binding is most apparent in the inner ear hair cells, leading to ototoxicity.

The m.1555A>G variant is strongly associated with AIO and has a reported prevalence of 0.2% (~1 in 500).[7] It has previously been suggested that genetic testing should be used in children requiring aminoglycosides to prevent hearing loss and that this approach would be cost-effective when balanced against the costs of lifelong deafness.[3,7] In many centers, children with cystic fibrosis are tested for the variant at diagnosis as it is expected that these individuals will require aminoglycoside

For peer review only - http://bmjopen.bmj.com/site/about/guidelines.xhtml

antibiotics at some stage in their lives. This is typically undertaken by a validated genotyping assay in an accredited diagnostic laboratory, a strategy which takes approximately 3-4 days to return a result. Understandably, this testing strategy is not suitable for use in clinical settings where administration of antibiotics is required within an hour of the decision to start treatment with antibiotics.

Currently there is an inability to test for the m.1555A>G variant in the acute setting such as babies admitted to the neonatal intensive care unit (NICU), representing approximately 90,000 admissions per year in the UK alone. By introducing a simple genetic point of care test (POCT) with a result in under 30 minutes, we have the potential to avoid approximately 180 cases of severe/profound irreversible deafness every year. Those babies with the m.1555A>G variant could be prescribed an equally effective second line antibiotic, such as a cephalosporin. The UK NICE guidance for early onset neonatal infection recommends a combination of gentamicin and benzylpenicillin as first-line therapy not due to superiority over other agents on an individual patient level, but because that regimen has a narrower spectrum of activity and therefore does not readily contribute towards the development of resistant bacterial pathogens.[4]

Alongside an industry partner, genedrive™, we have a developed a small and robust thermocycler platform for rapid point-of-care diagnostic testing of the m.1555A>G variant which has been CE certified for clinical use. This protocol describes a multi-centre prospective observational trial to assess the implementation of the POCT in the clinical setting.

2. Methods

2.1 Study Design

Investigator-initiated, multi-centre, prospective-observational, implementation trial. Two study sites, both of which are recruiting and experimental.

2.2 Primary Objective

For peer review only - http://bmjopen.bmj.com/site/about/guidelines.xhtml

To critically assess the performance of a newly developed genetic POCT for use on the NICU and measure whether the clinical teams can integrate the test into their clinical practice without disrupting normal standard of care, facilitating tailored antibiotic prescribing.

2.3 Secondary Objectives

Exploratory objectives include an assessment of the wider impact of the implementation, including impact on resources and clinical timelines. We will also assess instances where the assay was not used and consider reasons for deviation. The reliability of the assay itself will also be measured through regular monitoring of results, assessment of test failures and confirmation against gold-standard genotyping.

2.4 Study Centres and Time Schedule

Participants will be recruited from two large UK based NICUs in the UK, Manchester University NHS Foundation Trust (MFT) and Liverpool Women's NHS Foundation Trust (LWH). Both sites will recruit participants and conduct the trial. It should be noted that there is considerable variation in antimicrobial prescribing practices both between and within different countries. The type of antibiotics used, dosing regimens, and monitoring protocols can vary between different nations, cities, and institutions.[8] Both units in this study use a combination of a beta-lactam and an aminoglycoside as first line therapy for early onset neonatal infection. Based on historical data, the majority of admissions to both units will be screened for infection and, as such, considered for antibiotic therapy. The trial began in January 2020 and the last participant was recruited in November 2020. The study continued despite the SARS-CoV-2 pandemic.

2.5 Primary Outcome

The primary outcome is the number of neonates who are successfully tested for the m.1555A>G variant via this novel POCT genetic test as a proportion of all babies who receive aminoglycoside antibiotics on the two participating NICUs.

For peer review only - http://bmjopen.bmj.com/site/about/guidelines.xhtml

2.6 Secondary Outcomes

This trial will also assess several secondary outcomes including the total number of neonates identified with the m.1555A>G genetic variant (positive result) as a proportion of the study population. An assessment of resource impact will be undertaken, including a health economic analysis of additional staff time to secure sample and testing and costs associated with alternative antibiotic prescribing for any positive tests. We will record the number of neonates where testing was not undertaken and the rationale for this. Correlation of the POCT result with the current in-house reference assay will be assessed and any test fails will be reported. In the acute neonatal setting, delivering antibiotics in a timely manner is critical and this should ideally be within an hour after admission. As such, we will assess whether the time to antibiotic administration after admission is impacted by implementation of the genetic POCT. A reference time to antibiotic was measured over a month-long period before implementation.

2.7 Inclusion criteria

All babies admitted to NICU across the two participating sites, commencing from the trial start date. This includes babies admitted directly from delivery suite, midwifery led units or transferred from another neonatal unit. At the LWH site, babies who are screened for infection on the NICU but then transferred to external wards (not formally admitted to NICU) will also be included. It is a clinical decision whether the babies are being screened for infection as part of their assessment, reflecting the real-world, pragmatic, nature of the trial. This also reflects the variation in admission procedures between the two trial sites where pathways differ due to local practices.

Given that all babies admitted to NICU are eligible for recruitment, some of those recruited will not go onto receive antibiotic therapy. If the baby did not go onto receive antibiotics, their data will still form part of the dataset for analysis.

2.8 Exclusion Criteria

For peer review only - http://bmjopen.bmj.com/site/about/guidelines.xhtml

Babies requiring antibiotics immediately, as determined by the admitting clinician, with already established intravenous (IV) access. These exclusion criteria recognize the potential urgency of any decision to deliver antibiotics. If IV access has not been achieved, then this may provide time to run the assay. We expect that only a small proportion of admissions would be excluded based on these criteria.

2.9 Training

Training of a minimum of 80% of relevant nursing and medical staff within the two NICUs will commence in the 6 months prior to study start. This figure has been chosen, based on guidance from senior NICU clinicians and nursing staff, to ensure that there will always be staff members on-shift who are trained to undertake the assay throughout the study.

Training will include practical use and interpretation of the assay, with Standard Operating Procedures for use integrated into the standard admission procedure. A "train the trainer" approach will be adopted, where a number of experienced NICU research nurses plus additional clinical nursing staff identified as "super-users" will receive training directly from representatives of the device manufacturer. These designated super-users will then cascade training to the remaining nursing and medical staff within the two neonatal services.

Ongoing refresher training and online resources will be made available throughout the trial. A training log will be maintained detailing staff competency including dates of training, in order to identify any correlation of assay performance with increasing training numbers. NICU research nurses and co-investigators will be fully Good Clinical Practice (GCP) competent. A local, named co-investigator (neonatal consultant) will be responsible for clinical staff performing any duties in accordance with the protocol, GCP and local requirements.

2.10 Testing Process & Assessment

For peer review only - http://bmjopen.bmj.com/site/about/guidelines.xhtml

Participants will be recruited as they are admitted or reviewed by the NICU team. Given the acute nature of the project and the timeframes involved, there is no opportunity to undertake a formal pre-trial screening assessment or prospective consenting process. All babies admitted to NICU or screened for infection by the NICU team will be deemed eligible for recruitment unless there is a clinical decision by the responsible admitting clinician that the exclusion criterion is met and the individual requires immediate antibiotic therapy, without delay.

Following recruitment, the time that a decision was made to prescribe antibiotics represents time 0 (Figure 1). If antibiotics were not prescribed, then time 0 is represented by the time of admission. The genetic POCT is performed from a buccal swab. When babies are admitted to NICU several swabs are already taken at admission. Where babies are recruited to the study, the m.1555A>G buccal swab will be performed alongside these normal admission processes. The assay will be commenced, and normal practice will continue. Once the assay result is available, it can be used to then undertake personalized antibiotic prescribing, avoiding aminoglycoside antibiotics if the m.1555A>G variant is detected. The CE certified assay will be integrated into the admission pathways at both centres and clinicians will not be blinded to the results.

As part of the study the following variables will be recorded; participant demographics, admission time, time of decision to prescribe, time swab taken, time assay started, time of assay result, assay result, time of antibiotic prescription, antibiotics prescribed. Where a positive result is identified and antibiotic therapy is required, an alternative antibiotic regimen (standard non-gentamicin pathway as per hospital guidelines) will be prescribed and a referral should be made to clinical genetics for familial cascade testing. Where there is a test-fail, standard of care should be followed, i.e the baby should be administered gentamicin if clinically indicated. Negative results will not be formally reported to the parents. However, all parents will be provided with information on the study in their information pack, allowing them to contact the study team for further information if they wish.

For peer review only - http://bmjopen.bmj.com/site/about/guidelines.xhtml

Assay performance will be assessed throughout the study, with at least 10% of samples each month tested via gold-standard sequencing. Any positive samples will also be confirmed as per standard clinical pathways. Any divergence between the POCT and gold-standard methodology will be reviewed by the trial management committee.

Data collection will be undertaken contemporaneously by the clinical staff and any missing data will be collected by the NICU research nurses. Data will be entered into an electronic database by the clinical team and a study ID will be generated. The participants will subsequently be anonymized to the research team. If an individual is not tested for any reason (e.g. test overlooked, staff availability or the individual meets the exclusion criteria), these incidences will be captured retrospectively by NICU research nurses by checking daily admission records and the reasons will be documented.

2.11 Samples Size

The sample size (approximately 900 babies) is estimated based upon the expected number of admissions across both clinical sites over an approximate 10-month trial period. To achieve a statistical power of 90% with a 5% significance level, a margin of 0.5 (in Cohen's d, which equates to about 7 minutes difference given the standard deviation in the control sample) and a control sample of N = 50, a test sample size of 112 is required. With the forecast test sample size of 900, statistical power should be around 95% to detect variance in clinical timings before and after implementation.

2.12 Statistical Analysis

For the primary outcome, simple descriptive statistics will be used to provide a metric for the proportion of babies tested for the variant over the number who were ultimately prescribed aminoglycoside antibiotics.

Regarding the secondary outcome measures, the objective is to establish that the introduction of the POCT does not significantly impact the time between admission to NICU and the prescription of antibiotics for acute admissions. For the purposes of this analysis, those babies receiving antibiotics

For peer review only - http://bmjopen.bmj.com/site/about/guidelines.xhtml

within the first 2 hours of admission, representing acute antibiotic administration, will be compared. The burden of proof rests on the research hypothesis, meaning the null hypothesis is that the new POCT does impact this quantity. This is a so-called non-inferiority significance test. The quantity of interest is the mean difference between the admission to ICU and prescription of antibiotics timestamps in two situations: with and without the POCT test $(\mu_c,\mu_{t)})$. The null hypothesis is that the difference between these quantities is greater than or equal to some prespecified margin (Δ) i.e. H_0: $\mu_c - \mu_t \geq \Delta; H_A: U_t - U_c < \Delta$. A type I error here is concluding the time durations are equivalent when in fact the time to antibiotic with the POCT is greater.

The two samples are of unequal size, with the control ('before implementation') sample having about n_c=50, and a forecasted test sample size of around n_t=900. We also have no insight into population variance and cannot assume they are the same in both groups (it may for example take some staff members longer to carry out the new test than others). As such, a one-sided Welch t-test will be used to reject or not reject the null hypothesis.

Throughout the study we anticipate that there will be iterative updates to the POCT technology, as avenues for optimization emerge because of our findings from real-world implementation. The timings of these updates will be recorded and separate post-hoc statistical analysis will be undertaken to assess the impact of each update.

2.13 Study Organisation

Two groups have been convened to oversee the trial, a management group, and a steering committee. The Trial Management Group convenes monthly to ensure all practical details of the trial are progressing well, within the agreed milestones, and is led by the study Chief Investigator. The trial management group will act as the data monitoring committee. Membership is independent of the sponsors. The data management team will have access to anonymized data and will review assay performance and trial conduct on a monthly basis- specifically the concordance with gold standard.

For peer review only - http://bmjopen.bmj.com/site/about/guidelines.xhtml

All data will be analyzed by an independent statistical team who report monthly to the trial management group. Any amendments to the protocol will be communicated to the REC, funding agency and the trials registry.

The Trial Steering Committee meets on a 6 monthly basis to monitor the performance of the trial against the agreed project plan and advise on scientific and technical aspects of the project. The membership is independent of the Sponsor and Investigators.

A Stakeholders group has also been assembled to advise on the design and dissemination of the study intervention in the interests of the intended end users and participants. This membership includes personnel from key clinical services (neonatology and paediatric audiology), commissioners (NHS England) and newborn and hearing loss charities.

2.14 Patient and Public Involvement

Involvement of parent and public representatives has been a critical component of the development of this trial protocol. Parent representatives are involved in both the trial management groups, stakeholders committee and a separate Public and Patient Involvement and Engagement (PPIE) panel, providing both neonatal care and/or hearing loss experience. The PPIE panel was involved in the development of both the protocol and ethics application. The PI and co-investigators presented early versions of the texts to the PPIE panel and any potentially contentious issues were discussed in more detail. The insights gained from these meetings were used to refine the applications prior to submission.

2.15 Ethics and Adverse Events

This is the world's first trial, that we are aware of, of a genetic POCT to personalize medicine in the acute neonatal setting. As such, there have been several ethical challenges, mainly performing DNA analysis without qualifying consent. Prospective consent is not possible given the acute nature of the admission, therefore we sought permission to proceed with the POCT included as part of the standard

For peer review only

of care. The parents could then opt-out of the data being used as part of the study. This ethical process has been described in detail previously.[9] Approval was provided by the Research Ethics Committee (REC) and the Human Research Authority (HRA) in August 2019. We will record any adverse event related to the sample collection procedure or directly related to the testing process.

2.16 Dissemination of Results

The results of the study will be submitted to international peer-reviewed scientific journals, irrespective of their outcome and presented as per best practice STROBE guidelines for observational trials.

3. Discussion

To our knowledge, the PALOH study represents the first trial of a genetic POCT designed to alter management in an acute, time-sensitive setting. Successful implementation could avoid up to 180 cases of aminoglycoside induced ototoxicity in the UK each year, and many more worldwide. However, this study has implications beyond this gene-drug pair. The anticipated results will indicate whether genetic technology can be implemented in the acute setting more broadly, a hitherto untested concept. The unprecedented nature of this work led to several practical and ethical challenges in the design of the protocol.

Several studies have previously reported that most clinicians are not confident with the interpretation and implementation of genetic data.[10–12] With this in mind, we ensured that the assay implemented as part of this trial was as straightforward to interpret as possible, providing a binary readout for the presence or absence of the m.1555A>G variant. However, even if an assay is simple to operate and interpret, we recognized that the imposition of any alien process in such a critical time-period could impact adoption. We therefore designed this protocol to include a comprehensive training strategy prior to the trial commencing on both units. This significant undertaking ensures that over 500 health care professionals, greater than 80% of all staff, have exposure to the system before implementation.

For peer review only - http://bmjopen.bmj.com/site/about/guidelines.xhtml

Due to CE certification requirements for medical devices in the European Union (EU), any new system must be validated prior to clinical implementation. For a technology which has been designed for use in the acute setting, this requirement poses unique challenges. Although we were able to thoroughly assess the technology in the pre-clinical laboratory setting, satisfying the requirements for CE certification, it was not possible to perform analytical validation in its intended use setting, the NICU. This was because any result generated in this setting would be actionable and could impact management, precipitating an ethical dilemma. As a result, we determined that the most appropriate strategy was to regularly review the performance of the assay following implementation and introduce any updates to the training or technology as required. We have therefore made clear in our analysis plan that outcome statistics will be reported for the study as a whole and for each time-period post-update, if any such updates are necessary.

Given the acute context in which the PALOH trial is taking place, it was recognised that it would not be practicable for informed consent to be gained prospectively. Newborns admitted to NICU are, by definition, acutely unwell and it is likely to represent one of the most stressful time in a parent's life. As such, it was determined that asking for consent to perform this genetic test immediately after birth would be inappropriate. Following discussion with stakeholders including parents, neonatologists, and geneticists, ethical approval was sought based on the POCT being included as part of the standard package of care offered to the acutely unwell neonate, for which broad parental consent is typically provided, or if unavailable, is undertaken in the best interest of the child.[13] Our PPIE panel were particularly helpful during this process, providing a forum to discuss the various consenting options where we could consider relative benefits and costs of each strategy.

It should be noted that there is considerable variation in antimicrobial prescribing practices both between and within different countries. The type of antibiotics used, dosing regimens and monitoring protocols can vary between different nations, cities, and institutions. The reasons for this are multifactorial, associated with historical practice, the adoption of clinical guidelines, and economic

For peer review only - http://bmjopen.bmj.com/site/about/guidelines.xhtml

considerations.[14–16] Where there are national guidelines, such as those produced by NICE, one would expect relative consistency in prescribing practice. However, where practice is less guideline driven, there may be more variation. This is exemplified by a retrospective cohort study of 52,061 infants from 127 NICUs across California, which found that antibiotic use varied between centers by up to 40-fold.[15] Variability in prescribing practice in the UK is likely to be less pronounced, given the more guideline driven, nationalised nature of its healthcare system. As such, health economic cases for the utilization of POCT systems, such as the one described here, will vary depending on the wider healthcare context and should be formally examined locally.

Neonates are tested for the variant as part of their admission process via a buccal swab, the variant details then used to inform prescribing and data is subsequently gathered and analysed. Parents will be provided information regarding the trial by the NICU team as part of an existing parental information pack. They would have the right to ask for their child's data to be removed from the study at that stage. This provides a mechanism to opt-out prior to the analysis stage although, critically, by this point their child will have already had tailored antibiotic prescribing based on the presence or absence of the variant.

To our knowledge this represents the first example of such a consenting model being used in a trial involving genetic testing. In the summer of 2019, an application for ethical approval was submitted to the Health Research Authority Research Ethics Service (RES), proposing that the importance of this genetic test and the potential benefits warranted approval of an opt-out consenting model. After consideration, the REC approved the PALOH trial design in full pending HRA approval, making it the first trial of a genetic POCT to alter management in the acute setting. However, in the week following the REC meeting the PALOH study team received notice that the HRA were withholding approval as they had concerns that the study was in violation of the Human Tissue Act (2004) (HT Act). Specifically,

For peer review only - http://bmjopen.bmj.com/site/about/guidelines.xhtml

the HRA felt that the trial design did not meet the legal definition of "qualifying consent" as outlined in the HT Act.

Following protracted ethical and legal discussion, the trial design was approved following the agreement that the act of performing the initial test represents a clinical decision, rather than a research question. This approach was then permissible under Schedule 4 Part 2 of the HT Act which outlines that "the medical diagnosis or treatment of the person whose body manufactured the DNA" is an excepted purpose for DNA analysis without formal consent.[9] We believe that the PALOH study represents the first time this ethicolegal issue has been considered. As such, our methodology may be of value to future clinical academics exploring the application of similar pharmacogenetic biomarkers.

The anticipated results of the PALOH trial will assess whether a genetic POCT can be used to tailor antibiotic prescribing in the acute NICU setting. This will be of value not only in avoiding AIO but also in optimising management in other disease areas, as the relevance of genetics in everyday clinical practice continues to develop. We believe that this trial methodology and the ethical issues outlined in this work will be of value when researchers are designing similar studies in the future.

4. Trial Status

The PALOH Study has closed to recruitment and analysis is ongoing. Recruitment began in January 2020 and completed in November 2020.

5. Declarations

Ethics Approval: Health Research Authority (HRA) and Health and Care Research Wales (HCRW) approval was granted by the Research Ethics Committee at Liverpool Women's Hospital on the 23rd of September 2019. REC Reference 19/NW/0400.

Consent for Publication: Consent for publication is not applicable as there are no identifying images or other personal details of participants presented in this protocol manuscript.

For peer review only - http://bmjopen.bmj.com/site/about/guidelines.xhtml

For peer review only

Competing Interests: SA, LK and GM are employees of genedrive. The other authors report no competing interests.

Data: All results of this trial will be submitted for peer reviewed publication. Final data will be submitted to an open access repository on publication of the final trial manuscript.

Author Statements: JHM and WGN developed the concept for the study. SA, LK and GM designed and optimized the assay for implementation. All authors contributed to the development of the protocol led by AM and MT and the Manchester Foundation Trust and Liverpool Women's Hospital sites, respectively. KH and NB manage the data collection at each clinical site. RM is research manager for PALOH. DS, PR, and PW are involved in data analysis. RMc and FU have designed and undertaken stakeholder interviews which contribute to the design of the protocol. JG and RC, along with RM, have overseen the PPIE process. RB, IB and PW have advised on the implementation process and broader study design. All authors are responsible for either the recruitment of participants, data collection or data analysis. All authors read and approved the final version of this manuscript.

Acknowledgements: The authors would like to thank the patients and family members who took part in the study. We would like to acknowledge the huge efforts of the nurses, physicians and administrative staff at both study sites who have been instrumental in developing the project. We are very grateful to the parents and public representatives who sat on the PPIE committee and advised on the development of the project.

Funding: Funding for the project was provided via an NIHR i4i program award (Grant Number: II-LB-0417-20002), with additional support from the Manchester NIHR Biomedical Research Centre, Hearing Health Theme (IS-BRC-1215-20007). Pilot data support from Action on Hearing Loss Flexi grant F67_Newm.

Figure Legends

Figure 1. Flow diagram demonstrating the admission, testing, and prescribing process in the PALOH Trial. The time that a decision was made to prescribe antibiotics represents time 0. If antibiotics were not prescribed, then time 0 is represented by the time of admission.

For peer review only - http://bmjopen.bmj.com/site/about/guidelines.xhtml

References

1 Schacht J, Talaska AE, Rybak LP. Cisplatin and Aminoglycoside Antibiotics: Hearing Loss and Its Prevention. *Anat Rec (Hoboken)* 2012; **295**: 1837–1850.

For peer review only - http://bmjopen.bmj.com/site/about/guidelines.xhtml

2 Lanvers-Kaminsky C, Ciarimboli G. Pharmacogenetics of drug-induced ototoxicity caused by aminoglycosides and cisplatin. *Pharmacogenomics* 2017; **18**: 1683–1695.

3 Bitner-Glindzicz M, Rahman S. Ototoxicity caused by aminoglycosides. *BMJ* 2007; **335**: 784–785.

4 National Institute for Health and Care Excellence. CG149. Neonatal infection (early onset): antibiotics for prevention and treatment. 2012.

5 Prayle A, Watson A, Fortnum H, Smyth A. Side effects of aminoglycosides on the kidney, ear and balance in cystic fibrosis. *Thorax* 2010; **65**: 654–658.

6 Prezant TR, Agapian JV, Bohlman MC, Bu X, Öztas S, Qiu W-Q *et al.* Mitochondrial ribosomal RNA mutation associated with both antibiotic–induced and non–syndromic deafness. *Nature Genetics* 1993; **4**: 289–294.

7 Bitner-Glindzicz M, Pembrey M, Duncan A, Heron J, Ring SM, Hall A *et al.* Prevalence of Mitochondrial 1555A→G Mutation in European Children. *N Engl J Med* 2009; **360**: 640–642.

8 Gkentzi D, Dimitriou G. Antimicrobial Stewardship in the Neonatal Intensive Care Unit: An Update. *Curr Pediatr Rev* 2019; **15**: 47–52.

9 McDermott JH. Genetic testing in the acute setting: a round table discussion. *Journal of Medical Ethics* 2020; **46**: 531–532.

10 Christensen KD, Vassy JL, Jamal L, Lehmann LS, Slashinski MJ, Perry DL *et al.* Are physicians prepared for whole genome sequencing? a qualitative analysis. *Clin Genet* 2016; **89**: 228–234.

11 Hauser D, Obeng AO, Fei K, Ramos MA, Horowitz CR. Views Of Primary Care Providers On Testing Patients For Genetic Risks For Common Chronic Diseases. *Health Aff (Millwood)* 2018; **37**: 793–800.

12 Selkirk CG, Weissman SM, Anderson A, Hulick PJ. Physicians' preparedness for integration of genomic and pharmacogenetic testing into practice within a major healthcare system. *Genet Test Mol Biomarkers* 2013; **17**: 219–225.

13 Coulson-Smith P, Lucassen A. Using biomarkers in acute medicine to prevent hearing loss: should this require specific consent? *Journal of Medical Ethics* 2020; **46**: 536–537.

14 Buke C, Hosgor-Limoncu M, Ermertcan S, Ciceklioglu M, Tuncel M, Köse T *et al.* Irrational use of antibiotics among university students. *J Infect* 2005; **51**: 135–139.

15 Schulman J, Dimand RJ, Lee HC, Duenas GV, Bennett MV, Gould JB. Neonatal Intensive Care Unit Antibiotic Use. *Pediatrics* 2015. doi:10.1542/peds.2014-3409.

16 Lansang MA, Lucas-Aquino R, Tupasi TE, Mina VS, Salazar LS, Juban N *et al.* Purchase of antibiotics without prescription in Manila, the Philippines. Inappropriate choices and doses. *J Clin Epidemiol* 1990; **43**: 61–67.

For peer review only

For peer review only - http://bmjopen.bmj.com/site/about/guidelines.xhtml

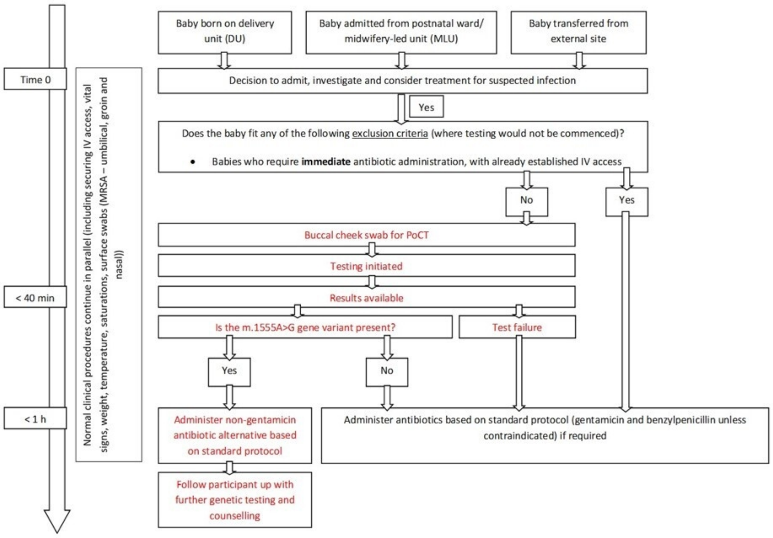

Figure 1. Flow diagram demonstrating the admission, testing, and prescribing process in the PALOH Trial. The time that a decision was made to prescribe antibiotics represents time 0. If antibiotics were not prescribed, then time 0 is represented by the time of admission.

100x60mm (300 x 300 DPI)

STROBE Statement—checklist of items that should be included in reports of observational studies

	Item No	Recommendation	Page No
**Title and abstract**	1	(*a*) Indicate the study's design with a commonly used term in the title or the abstract	1
		(*b*) Provide in the abstract an informative and balanced summary of what was done and what was found	2
**Introduction**			
Background/rationale	2	Explain the scientific background and rationale for the investigation being reported	5
Objectives	3	State specific objectives, including any prespecified hypotheses	6
**Methods**			
Study design	4	Present key elements of study design early in the paper	6
Setting	5	Describe the setting, locations, and relevant dates, including periods of recruitment, exposure, follow-up, and data collection	7
Participants	6	(*a*) *Cohort study*—Give the eligibility criteria, and the sources and methods of selection of participants. Describe methods of follow-up<br>*Case-control study*—Give the eligibility criteria, and the sources and methods of case ascertainment and control selection. Give the rationale for the choice of cases and controls<br>*Cross-sectional study*—Give the eligibility criteria, and the sources and methods of selection of participants	7 & 8
		(*b*) *Cohort study*—For matched studies, give matching criteria and number of exposed and unexposed<br>*Case-control study*—For matched studies, give matching criteria and the number of controls per case	N/A
Variables	7	Clearly define all outcomes, exposures, predictors, potential confounders, and effect modifiers. Give diagnostic criteria, if applicable	7
Data sources/ measurement	8*	For each variable of interest, give sources of data and details of methods of assessment (measurement). Describe comparability of assessment methods if there is more than one group	*9*
Bias	9	Describe any efforts to address potential sources of bias	13
Study size	10	Explain how the study size was arrived at	9 & 10
Quantitative variables	11	Explain how quantitative variables were handled in the analyses. If applicable, describe which groupings were chosen and why	10
Statistical methods	12	(*a*) Describe all statistical methods, including those used to control for confounding	10
		(*b*) Describe any methods used to examine subgroups and interactions	N/A
		(*c*) Explain how missing data were addressed	N/A
		(*d*) *Cohort study*—If applicable, explain how loss to follow-up was addressed<br>*Case-control study*—If applicable, explain how matching of cases and controls was addressed<br>*Cross-sectional study*—If applicable, describe analytical methods taking account of sampling strategy	N/A
		(*e*) Describe any sensitivity analyses	N/A

For peer review only - http://bmjopen.bmj.com/site/about/guidelines.xhtml

**Results**			
Participants	13*	(a) Report numbers of individuals at each stage of study—eg numbers potentially eligible, examined for eligibility, confirmed eligible, included in the study, completing follow-up, and analysed	Awaited
		(b) Give reasons for non-participation at each stage	Awaited
		(c) Consider use of a flow diagram	Awaited
Descriptive data	14*	(a) Give characteristics of study participants (eg demographic, clinical, social) and information on exposures and potential confounders	Awaited
		(b) Indicate number of participants with missing data for each variable of interest	Awaited
		(c) *Cohort study*—Summarise follow-up time (eg, average and total amount)	Awaited
Outcome data	15*	*Cohort study*—Report numbers of outcome events or summary measures over time	Awaited
		Case-control study—Report numbers in each exposure category, or summary measures of exposure	Awaited
		Cross-sectional study—Report numbers of outcome events or summary measures	Awaited
Main results	16	(*a*) Give unadjusted estimates and, if applicable, confounder-adjusted estimates and their precision (eg, 95% confidence interval). Make clear which confounders were adjusted for and why they were included	Awaited
		(*b*) Report category boundaries when continuous variables were categorized	Awaited
		(*c*) If relevant, consider translating estimates of relative risk into absolute risk for a meaningful time period	Awaited
Other analyses	17	Report other analyses done—eg analyses of subgroups and interactions, and sensitivity analyses	Awaited
**Discussion**			
Key results	18	Summarise key results with reference to study objectives	Awaited
Limitations	19	Discuss limitations of the study, taking into account sources of potential bias or imprecision. Discuss both direction and magnitude of any potential bias	Awaited
Interpretation	20	Give a cautious overall interpretation of results considering objectives, limitations, multiplicity of analyses, results from similar studies, and other relevant evidence	Awaited
Generalisability	21	Discuss the generalisability (external validity) of the study results	Awaited
**Other information**			
Funding	22	Give the source of funding and the role of the funders for the present study and, if applicable, for the original study on which the present article is based	3

*Give information separately for cases and controls in case-control studies and, if applicable, for exposed and unexposed groups in cohort and cross-sectional studies.

Note: An Explanation and Elaboration article discusses each checklist item and gives methodological background and published examples of transparent reporting. The STROBE checklist is best used in conjunction with this article (freely available on the Web sites of PLoS Medicine at http://www.plosmedicine.org/, Annals of Internal Medicine at http://www.annals.org/, and Epidemiology at http://www.epidem.com/). Information on the STROBE Initiative is available at www.strobe-statement.org.

For peer review only - http://bmjopen.bmj.com/site/about/guidelines.xhtml
